# A Multi-Species TaqMan PCR Assay for the Identification of Asian Gypsy Moths (*Lymantria* spp.) and Other Invasive Lymantriines of Biosecurity Concern to North America

**DOI:** 10.1371/journal.pone.0160878

**Published:** 2016-08-11

**Authors:** Donald Stewart, Reza Zahiri, Abdelmadjid Djoumad, Luca Freschi, Josyanne Lamarche, Dave Holden, Sandra Cervantes, Dario I. Ojeda, Amélie Potvin, Audrey Nisole, Catherine Béliveau, Arnaud Capron, Troy Kimoto, Brittany Day, Hesther Yueh, Cameron Duff, Roger C. Levesque, Richard C. Hamelin, Michel Cusson

**Affiliations:** 1 Laurentian Forestry Centre, Canadian Forest Service, Natural Resources Canada, Quebec City, Quebec, Canada; 2 Canadian Food Inspection Agency, Ottawa Plant Laboratory, Entomology Unit, Ottawa, Ontario, Canada; 3 Institut de Biologie Intégrative et des Systèmes, Université Laval, Quebec City, Quebec, Canada; 4 Canadian Food Inspection Agency, Burnaby, British Columbia, Canada; 5 Department of Forest and Conservation Sciences, Faculty of Forestry, University of British Columbia, Vancouver, British Columbia, Canada; 6 Canadian Food Inspection Agency, National Headquarters, Ottawa, Ontario, Canada; University of Helsinki, FINLAND

## Abstract

Preventing the introduction and establishment of forest invasive alien species (FIAS) such as the Asian gypsy moth (AGM) is a high-priority goal for countries with extensive forest resources such as Canada. The name AGM designates a group of closely related *Lymantria* species (Lepidoptera: Erebidae: Lymantriinae) comprising two *L*. *dispar* subspecies (*L*. *dispar asiatica*, *L*. *dispar japonica*) and three closely related *Lymantria* species (*L*. *umbrosa*, *L*. *albescens*, *L*. *postalba*), all considered potential FIAS in North America. Ships entering Canadian ports are inspected for the presence of suspicious gypsy moth eggs, but those of AGM are impossible to distinguish from eggs of innocuous *Lymantria* species. To assist regulatory agencies in their identification of these insects, we designed a suite of TaqMan^®^ assays that provide significant improvements over existing molecular assays targeting AGM. The assays presented here can identify all three *L*. *dispar* subspecies (including the European gypsy moth, *L*. *dispar dispar*), the three other *Lymantria* species comprising the AGM complex, plus five additional *Lymantria* species that pose a threat to forests in North America. The suite of assays is built as a “molecular key” (analogous to a taxonomic key) and involves several parallel singleplex and multiplex qPCR reactions. Each reaction uses a combination of primers and probes designed to separate taxa through discriminatory annealing. The success of these assays is based on the presence of single nucleotide polymorphisms (SNPs) in the 5’ region of mitochondrial cytochrome c oxidase I (COI) or in its longer, 3’ region, as well as on the presence of an indel in the “FS1” nuclear marker, generating North American and Asian alleles, used here to assess Asian introgression into *L*. *dispar dispar*. These assays have the advantage of providing rapid and accurate identification of ten *Lymantria s*pecies and subspecies considered potential FIAS.

## Introduction

For countries dominated by forested land such as Canada, expanding global trade has increased the risks of introduction and establishment of forest invasive alien species (FIAS). This situation has called for heightened vigilance on the part of plant protection authorities and a strengthening of measures taken to prevent the accidental introduction of unwanted alien pests. Some FIAS represent a greater threat than others, and the Asian gypsy moth (AGM) ranks high on that list. For regulatory purposes, what is referred to as AGM is a group of closely related *Lymantria* (Lepidoptera: Erebidae: Lymantriinae) moths comprising two *L*. *dispar* subspecies (*L*. *dispar asiatica* Vnukovskij and *L*. *dispar japonica* (Motschulsky)) and three other *Lymantria* species (*L*. *umbrosa* (Butler), *L*. *albescens* Hori and Umeno, and *L*. *postalba* Inoue [1–3]. The European gypsy moth (EGM), *L*. *dispar dispar* (Linnaeus), is already established in North America following an accidental introduction in Massachusetts in 1869 [4]. Although the EGM has since become a very serious pest of hardwoods in parts of the United States and Canada, its females are flightless and individuals of this subspecies have a narrower host range than their Asian *L*. *dispar* counterparts, which have >500 known hosts and whose females are flight-capable, thereby facilitating their spread following an accidental introduction [5]. The other three species making up the AGM group (*L*. *umbrosa*, *L*. *postalba*, *L*. *albescens*) form a clade with *L*. *dispar* [6] and were once considered *L*. *dispar* subspecies [1]. Their host ranges differ from those of *L*. *dispar asiatica* and *L*. *dispar japonica* but they are considered a threat to some parts of North America; for this reason, they are regulated in Canada [2,7]. In addition, at least five other *Lymantria* species pose a threat to forests in Canada and the United States: *L*. *monacha* (Linnaeus), *L*. *fumida* Butler, *L*. *mathura* Moore, *L*. *xylina* Swinhoe and *L*. *lucescens* (Butler). The first two species are conifer defoliators while the last three are broadleaf defoliators [1]. For an assessment of the phylogenetic relationships among these species/subspecies, the reader is referred to earlier studies by other groups [6,8–14].

In order to prevent the introduction of these insects into Canada, the Canadian Food Inspection Agency (CFIA) uses pheromone traps to monitor gypsy moths and conducts regular inspections of foreign vessels entering Canadian ports, where they search ships and cargo for moths, larvae and egg masses. Given that eggs of the above species and subspecies are virtually impossible to distinguish from one another and from other non-threatening *Lymantria* species (reliable identification requires rearing to the adult stage), we initiated the development of a rapid molecular suite of assays that can produce AGM diagnostic results hours after testing suspicious eggs, so that informed decisions can be made and actions taken to prevent accidental AGM introductions. Several other molecular diagnostic assays targeting AGMs and allied species have been developed by other groups, but most of these require either sequencing of amplified products followed by sequence comparisons [6,8–10,15,16] or implementation of one of various approaches involving amplification of markers, DNA digestion with restriction enzymes and gel electrophoresis [11–13,17–23]. Although some of these approaches have proven to be operationally useful, they do not provide rapid species/subspecies identification and may be of limited subspecies resolution. Recently, two rapid quantitative PCR (qPCR) TaqMan^®^ assays were reported for AGM diagnostics, but their scope is limited to assigning the unknown to either *L*. *dispar dispar* or *L*. *dispar asiatica/L*. *dispar japonica* [14,24].

Here we present a set of qPCR-based molecular assays that can identify all three *L*. *dispar* subspecies, the three additional *Lymantria* species comprising the AGM complex and all five other threatening *Lymantria* species (referred to here as “OTLS”) listed above. In cases where the unknown is identified as *L*. *dispar dispar*, one of the assays will also flag individuals displaying *L*. *dispar asiatica* introgression resulting from inter-subspecies hybridization; females of such hybrids can show strong flight capability [22,25], and regulators may decide to treat them as AGM. The suite of assays is built as a “molecular key” (analogous to a taxonomic key) and involves several parallel, singleplex and multiplex qPCR reactions. For any given reaction, it applies a combination of primers and probes, some of which feature locked nucleic acids (LNA probes [26]), designed to separate taxa through discriminatory annealing. The success of these assays is based on the presence of single nucleotide polymorphisms (SNPs) in the 5’ region of mitochondrial cytochrome c oxidase I (COI-5P; also known as the “barcode region”) or in its longer, 3’ region (COI-3P), as well as from an indel in the “FS1” nuclear marker, generating North American and Asian alleles (FS1 is an anonymous marker identified using a RAPD-PCR strategy [20]). These assays have the advantage of providing rapid (< 1 day) and accurate identification of ten *Lymantria s*pecies and subspecies, including several potential FIAS in Canada and the United States.

## Materials and Methods

### Sources of biological material and collection of data from databases

We built a panel of specimens comprising multiple individuals of the assays’ target species, plus closely related ones, including species that could be encountered and whose eggs could not be easily distinguished from those of target species. Specimens were provided by collaborators possessing taxonomic skills ensuring authentication of moth identification. To capture intraspecific genetic diversity, specimens from different geographic regions were used when available. The majority of the specimens used were dry adults taken from existing collections or fresh adults obtained from laboratory rearings established ~10 years ago. No specific permissions were required for collecting the remaining specimens, many of which were caught in pheromone traps or collected in the field as eggs or larvae in Canada (*L*. *dispar dispar)*. None of the insects used belong to an endangered species.

For assay development, we used as many DNA sequences per taxon as possible to cover the range of genetic diversity. This includes DNA sequences from all specimens in our collection as well as all publicly available DNA sequences for a given target gene. The complete list of DNA sequences used for assay development is provided in S1 Table.

### DNA extraction from adults

For some of the work conducted here, DNA was extracted from moths that were snap-frozen alive and held in the freezer until processed (specimens identified as “fresh adults” in Table 1). In these cases, adult males (wings and abdomen removed) were ground in liquid nitrogen and submitted to DNA extraction, using either the Qiagen Blood & Cell Culture DNA midi kit or the Qiagen DNeasy Blood & Tissue mini kit (Toronto, ON, Canada), following the manufacturer’s instructions. For DNA extraction from archival specimens (typically, two moth legs), we also used the Qiagen DNeasy Blood & Tissue mini kit, with the following modifications: incubation at step 3 was done overnight and elution at step 8/9 was done in a volume of 100 μL.

**Table 1:** Specimens of target species and closely related ones used for assay development and validation.

Specimen ID	Species/subspecies	Country of origin	Region	Year collected	Type of material	Supplier
**AGM complex**						
CFIA-LEP0246	*Lymantria albescens*	Japan	Kagoshima	2014	Dry adult	U. Jinbo
CFIA-LEP0247	*Lymantria albescens*	Japan	Kagoshima	2014	Dry adult	U. Jinbo
CFIA-LEP0306	*Lymantria albescens*	Japan	Ishigaki	2015	Dry adult	A. Schintlmeister
CFIA-LEP0307	*Lymantria albescens*	Japan	Okinawa	2003	Dry adult	A. Schintlmeister
AGM-0537	*Lymantria albescens*	Japan	Okinawa	2015	Dry adult	M. Kimura
AGM-0538	*Lymantria albescens*	Japan	Okinawa	2015	Dry adult	M. Kimura
AGM-0545	*Lymantria albescens*	Japan	Okinawa	2015	Dry adult	M. Kimura
AGM-0565	*Lymantria albescens*	Japan	Okinawa	2015	Dry adult	M. Kimura
AGM-0566	*Lymantria albescens*	Japan	Okinawa	2015	Dry adult	M. Kimura
AGM-0581	*Lymantria albescens*	Japan	Kumejima	2015	Dry adult	M. Kimura
AGM-0582	*Lymantria albescens*	Japan	Kumejima	2015	Dry adult	M. Kimura
AGM-0587	*Lymantria albescens*	Japan	Iriomote	2015	Dry adult	M. Kimura
AGM-0588	*Lymantria albescens*	Japan	Iriomote	2015	Dry adult	M. Kimura
AGM-0603	*Lymantria albescens*	Japan	Ishigaki	2015	Dry adult	M. Kimura
AGM-0604	*Lymantria albescens*	Japan	Ishigaki	2015	Dry adult	M. Kimura
AGM-0606	*Lymantria albescens*	Japan	Kohama	2015	Dry adult	M. Kimura
AGM-0607	*Lymantria albescens*	Japan	Kohama	2015	Dry adult	M. Kimura
AGM-0500	*Lymantria postalba*	Japan	Shikoku	2015	Dry adult	M. Kimura
AGM-0501	*Lymantria postalba*	Japan	Shikoku	2015	Dry adult	M. Kimura
AGM-0502	*Lymantria postalba*	Japan	Shikoku	2015	Dry adult	M. Kimura
AGM-0514	*Lymantria postalba*	Japan	Tsushima	2015	Dry adult	M. Kimura
AGM-0515	*Lymantria postalba*	Japan	Tsushima	2015	Dry adult	M. Kimura
AGM-0520	*Lymantria postalba*	Japan	Kyushu	2015	Dry adult	M. Kimura
AGM-0521	*Lymantria postalba*	Japan	Kyushu	2015	Dry adult	M. Kimura
AGM-0526	*Lymantria postalba*	Japan	Kyushu	2015	Dry adult	M. Kimura
AGM-0527	*Lymantria postalba*	Japan	Kyushu	2015	Dry adult	M. Kimura
AGM-0529	*Lymantria postalba*	Japan	Tanegashima	2015	Dry adult	M. Kimura
AGM-0530	*Lymantria postalba*	Japan	Tanegashima	2015	Dry adult	M. Kimura
AGM-0009	*Lymantria dispar asiatica*	Russia	-	2008	Dry adult	M. Kumachi
AGM-0018	*Lymantria dispar asiatica*	South Korea	Kangwon	2009	Dry adult	D. G. Holden
AGM-0024	*Lymantria dispar asiatica*	Russia	Primorsky Kray	2002	Dry adult	D. E. Nilsson/K. Larsen
AGM-0025	*Lymantria dispar asiatica*	Russia	Vladivostok	2008	Dry adult	N. Kummen
AGM-0026	*Lymantria dispar asiatica*	South Korea	Kangwon	2009	Dry adult	D. G. Holden
AGM-0027	*Lymantria dispar asiatica*	South Korea	Kangwon	2009	Dry adult	D. G. Holden
CFS-0003	*Lymantria dispar asiatica*	China	Tianjin	2007	Fresh adult	H. Nadel
CFS-0004	*Lymantria dispar asiatica*	Mongolia	-	-	Fresh adult	H. Nadel
CFS-0005	*Lymantria dispar asiatica*	China	Beijing	-	Fresh adult	M. Keena
CFS-0006	*Lymantria dispar asiatica*	China	Heilongjiang	-	Fresh adult	M. Keena
CFS-0007	*Lymantria dispar asiatica*	China	Liaoning	-	Fresh adult	M. Keena
CFS-0009	*Lymantria dispar asiatica*	Russia	Far east	-	Fresh adult	M. Keena
AGM-0001	*Lymantria dispar japonica*	Japan	Honshu	-	Dry adult	D. G. Holden
AGM-0015	*Lymantria dispar japonica*	Japan	Nagano	2012	Dry adult	D. G. Holden
AGM-0016	*Lymantria dispar japonica*	Japan	Chiba	2012	Dry adult	D. G. Holden
AGM-0017	*Lymantria dispar japonica*	Japan	Chiba	2012	Dry adult	D. G. Holden
AGM-0031	*Lymantria dispar japonica*	Japan	Yamanashi	2012	Dry adult	M. Tomonaga
AGM-0033	*Lymantria dispar japonica*	Japan	Yamanashi	2012	Dry adult	M. Tomonaga
AGM-0034	*Lymantria dispar japonica*	Japan	Chiba	2012	Dry adult	K. Ishizuka
CFS-0001	*Lymantria dispar japonica*	Japan	Honshu	-	Fresh adult	M. Keena
CFIA-LEP0450	*Lymantria dispar japonica*	Japan	Hokkaido	-	Dry adult	R. Zahiri
CFIA-LEP0451	*Lymantria dispar japonica*	Japan	Hokkaido	-	Dry adult	R. Zahiri
CFIA-LEP0453	*Lymantria dispar japonica*	Japan	Hokkaido	-	Dry adult	R. Zahiri
CFIA-LEP0452	*Lymantria umbrosa*	Japan	Hokkaido	2015	Dry adult	C. Hideyuki
**EGM and other *L*. *dispar* specimens of uncertain subspecies designation**^1^						
CFIA-LEP0035	*Lymantria dispar dispar*	Canada	British Columbia	2014	Trapped adult	CFIA^a^
CFIA-LEP0037	*Lymantria dispar dispar*	Canada	Newfoundland	2014	Trapped adult	CFIA
CFIA-LEP0145	*Lymantria dispar*	Lebanon	Aakkar	2008	Dry adult	A. Schintlmeister
CFIA-LEP0151	*Lymantria dispar*	Tajikistan	Varzob	2005	Dry adult	A. Schintlmeister
CFIA-LEP0195	*Lymantria dispar*	China	Xinjiang	2012	Dry adult	H. Wang
CFIA-LEP0234	*Lymantria dispar dispar*	France	Touraine	1993	Dry eggs	M.-J. Côté
CFIA-LEP0295	*Lymantria dispar*	Uzbekistan	Tashkent	2014	Dry adult	A. Schintlmeister
CFIA-LEP0299	*Lymantria dispar*	Russia	West Siberia	2015	Dry adult	A. Schintlmeister
CFIA-LEP0416	*Lymantria dispar*	Russia	Bashkortostan	2006	Dry adult	D. Shovkoon
CFIA-LEP0427	*Lymantria dispar dispar*	Russia	Crimea	2009	Dry adult	D. Shovkoon
CFIA-LEP0456	*Lymantria dispar*	Kazakhstan	Koram	2015	Dry adult	S. K. Korb
CFIA-LEP0465	*Lymantria dispar*	Russia	Volga	2012	Dry adult	S. K. Korb
CFIA-LEP0469	*Lymantria dispar*	Kazakhstan	Chuy	2015	Dry adult	S. K. Korb
CFIA-LEP0470	*Lymantria dispar*	Kazakhstan	Almaty	2015	Dry adult	S. K. Korb
CFIA-LEP0476	*Lymantria dispar*	Kyrgyzstan	Chuy	2015	Dry adult	S. K. Korb
CFIA-LEP0479	*Lymantria dispar*	Kyrgyzstan	Jalal-Abad	2015	Dry adult	S. K. Korb
CFIA-LEP0492	*Lymantria dispar dispar*	Czech Republic	Vyskov	2009	Dry adult	M. Rindos
CFIA-LEP0146	*Lymantria dispar*^2^	Iran	Kuh-e-Sendan	2005	Dry adult	A. Schintlmeister
CFIA-LEP0147	*Lymantria dispar*^2^	Iraq	Kirkuk	2005	Dry adult	A. Schintlmeister
AGM-0022	*Lymantria dispar dispar*^2^	Canada	Ontario	2001	Dry adult	D. G. Holden
AGM-0023	*Lymantria dispar dispar*	USA	Delaware	1997	Dry adult	P. Schaefer
AGM-0151	*Lymantria dispar dispar*	Canada	British Columbia	2015	Dry larvae	D. G. Holden
AGM-0162	*Lymantria dispar dispar*	Canada	British Columbia	2015	Dry eggs	D. G. Holden
CFS-0008	*Lymantria dispar*	Russia	Siberia	-	Fresh adult	M. Keena
CFS-0010	*Lymantria dispar*	Lithuania	-	-	Fresh adult	M. Keena
CFS-0011	*Lymantria dispar dispar*	Greece	-	-	Fresh adult	M. Keena
CFS-0012	*Lymantria dispar dispar*	USA	Connecticut	-	Fresh adult	M. Keena
**OTLS**						
AGM-0036	*Lymantria monacha*	Japan	Nagano	2012	Dry adult	M. Tomonaga
AGM-0067	*Lymantria monacha*	Japan	Nagano	2012	Dry adult	M. Tomonaga
CFS-0014	*Lymantria monacha*	Czech Republic	-	-	Fresh adult	M. Keena
CFIA-LEP0341	*Lymantria monacha*	China	Fujian	2014	Dry adult	A. Schintlmeister
AGM-0047	*Lymantria fumida*	USA	Delaware	1997	Dry adult	P. Schaefer
AGM-0002	*Lymantria mathura*	Japan	Nagano	2013	Dry adult	D. G. Holden
AGM-0050	*Lymantria mathura*	Japan	Nagano	2013	Dry adult	H. Hirano
AGM-0051	*Lymantria mathura*	Japan	Yamanashi	2012	Dry adult	M. Tomonaga
AGM-0052	*Lymantria mathura*	South Korea	Kangwon	2009	Dry adult	D. G. Holden
AGM-0054	*Lymantria mathura*	China	Yunnan	2014	Dry adult	D. G. Holden
CFIA-LEP0253	*Lymantria xylina*	Taiwan	Yilan	2011	Dry adult	S. Wu
CFIA-LEP0311	*Lymantria xylina*	Japan	Okinawa	-	Dry adult	A. Schintlmeister
AGM-0068	*Lymantria lucescens*	Japan	Nagano	2013	Dry adult	H. Hirano
CFIA-LEP0093	*Lymantria lucescens*	South Korea	North Gyeongsang	2005	Dry adult	S.-W. Choi
CFIA-LEP0094	*Lymantria lucescens*	South Korea	Kangwon	2005	Dry adult	S.-W. Choi
**Related species**						
AGM-0170	*Lymantria atemeles*	Thailand	Phayao	2011	Dry adult	P. Schaefer
AGM-0071	*Lymantria bantaizana*	Japan	Nagano	2013	Dry adult	S. Oshima
AGM-0164	*Lymantria concolor*	Thailand	Kanchanaburi	2011	Dry adult	P. Schaefer
AGM-0040	*Lymantria minimonis*	Japan	Nagano	2013	Dry adult	H. Hirano
AGM-0134	*Orgyia thyellina*	Japan	Okayama	2013	Dry adult	S. Miyake
AGM-0093	*Leucoma salicis*	Japan	Nagano	2012	Dry adult	S. Oshima
AGM-0096	*Leucoma salicis*	China	Beijing	1997	Dry adult	P. Schaefer
AGM-0138	*Arctornis I-nigrum*	Japan	Nagano	2012	Dry adult	M. Tomonaga
AGM-0139	*Arctornis I-nigrum*	South Korea	South Jeolla	2012	Dry adult	B.K. Byun
AGM-0107	*Calliteara abietis*	Japan	Yamanachi	2012	Dry adult	M. Tomonaga
AGM-0115	*Calliteara pudibunda*	Japan	Yamanachi	2012	Dry adult	M. Tomonaga
AGM-0127	*Cifuna locuples*	China	Yunnan	2014	Dry adult	D. G. Holden
AGM-0132	*Cifuna locuples*	South Korea	South Jeolla	2012	Dry adult	B.K. Byun
AGM-0121	*Euproctis similis*	Japan	Nagano	2012	Dry adult	S. Oshima

^1^ All specimens found in this section generated a positive amplification in the EGM assay (i.e., *Simplex assay 3*)

^2^ Used for FS1 validation only.

^a^ Canadian Food Inspection Agency

### DNA extraction from egg masses for direct PCR

DNA was extracted from fresh, post-diapause *Lymantria dispar dispar* egg masses (Insect Production Services, Natural Resources Canada, Sault Ste. Marie, ON) following the protocol described in [27]. Two eggs were added to 100 μL of 2x buffer (20 mM Tris, pH 8.3, 3 mM MgCl_2_, 100 mM KCl) in a 1.5 mL conical tube. Just prior to use, Tween 20 (final concentration 1% v/v) and 200 μg/mL Proteinase K (Life Technologies, Carlsbad, CA, USA) were added to the buffer. The samples were ground with a disposable micro-pestle (VWR, Radnor, PA, USA) and incubated at 55°C for 120 min, with a brief vortexing after the first 5 min of incubation. After centrifugation at 13,000 x ***g*** for 5 min, the supernatant was transferred to a new tube and heated at 99°C for 5 min to inactivate the Proteinase K. One hundred μL of dH_2_O was added to the sample to achieve a 1x dilution and 1 μL was used in qPCR.

### Marker amplification and sequencing

Universal primers used to amplify and sequence mitochondrial and nuclear genes from specimens of our *Lymantria* collection were designed based on publicly available sequences (GenBank, BOLD); primer sequences are provided in S2 Table. Mitochondrial genes initially selected for the development of our molecular assay included subunits of cytochrome c oxidase (COI and COII), NADH dehydrogenase (ND1, ND2 and ND6), ATP synthase (6 and 8) and cytochrome b oxidase whereas elongation factor-1 alpha (Ef-1α) was selected as a nuclear marker. DNA extraction, PCR amplification, and sequencing of the targeted molecular markers followed standard protocols [28–30]. PCR and sequencing of barcode region (COI-5P) generally used a single pair of primers that recovers a 658 bp region near the 5′ end of COI, including the 648 bp barcode region for the animal kingdom [31]. For older museum specimens, primer pairs designed to amplify smaller overlapping fragments (307 bp, 407 bp) were employed [32]. PCR reactions were performed directly on total DNA extracts (0.5–1 ng) in a 25 μL final volume. PCR conditions were as follows: initial denaturation step at 94°C for 2 min, followed by 35–40 cycles of denaturing (94°C, 1 min), annealing (43°C–55°C, 1 min, depending on primers and/or sample), extension (72°C, 1–2 min, depending on amplicon size) and a final extension of 72°C for 10 min. PCR products were used for direct Sanger sequencing; resulting sequences were compared to identify discriminant SNPs.

### Target-specific TaqMan-based real-time PCR assays

All molecular detection assays used in this study are based on TaqMan^®^ technology [33]. Primer and probe design was performed using Oligo Explorer v1.2 and Oligo Analyzer v1.2 (Gene Link, NY, USA). Primers (Table 2) and probes (Table 3) were designed to (i) minimize development of secondary structures and dimer formation at the 3’ end of primers (minimal interaction between primers and probes) and (ii) to ensure amplicon length does not exceed 200 bp. Interspecific SNPs were preferentially localized at the extreme 3’ end of the primers and the middle of the probes for maximum discriminatory effect. All primers and TaqMan probes were manufactured by Integrated DNA Technologies Inc. (IDT; Coralville, IA, USA). All assays were designed to work under the same thermocycling conditions.

**Table 2:** List of primers developed for DNA quantification and for each simplex, duplex and triplex assay, as identified in Fig 1. The first 3–4 letters of primer names designate the taxa being targeted (except for FS1, which designates the marker), while “F” and “R” refer to forward and reverse. In primer sequences, bold/underlined letters designate degenerate sites while italicized/underlined letters designate ARMS bases.

Assay	Assay #	Primer name	Primer sequence
*DNA quantif*.	Lymantriine general primers	Lep COI GEN F308-327	GAAAATGGAGC**I**GG**W**AC**W**GG
		Lep COI GEN R413-435	GCTCCTAAAAT**W**GA**I**GAAAT**W**CC
*Duplex 1*	1A	Lalb COI F3G/A 491–520	CAAATACCTTTATTTGTTTGAAGAGTA*A*GT
		Lalb COI R2C/A 562–590	GGTCAGTTAATAATATTGTAATAGCAC*A*C
	1B	AGM comp COI F3G/A 601–622	TACATCCTTTTTTGACCC**Y***A*C**R**
		AGM comp COI R701-719	TCCTCTTTCTTGGGAAATA
*Duplex 2*	2A	Ldaj COI F325-342	AGGATGAACTGTTTACCC
		Ldaj COI R428-454	GGTAGTAATAAAATTAATTGCTCCTAA
	2B	Lda COI3P F764-783	AGCTATCGGATTATTAGGATT
		Lda COI3P R803-832	GTATCAATATCTATACC**Y**ACAGTAAATATG
*Simplex 3*	3A	Ldd COI F488-513	GATCAAATACCTTTATTT**R**TTTGAAG
		Ldd COI R3A/G 574–607	GGATGTATTTAAATTTCGGTCAGTTAATAAT*G*TT
*Duplex 4*	4A-4B	FS1 F2-16	GATGGTGGGTGTCGT
		FS1 R176-200 (71–95)	GATTCATCTGATCCTGATAATTCAT
*Duplex 5*	5A	Lmon COI F183-205	GATTTGGAAATTGATTAGTACCT
		Lmon COI R274-300	TCTTGAAATTAAAAGGGTTAATGAT
	5B	Lfum COI F445-472	TATTACAACAATTATCAATATACGACTT
		Lfum COI R628-651	TGTTGATATAAAATAGGATCTCCA
*Triplex 6*	6A	Lmat COI F44-67	ACTTC**Y**CT*T*AGATT**R**TTAATTCGC
		Lmat COI R214-230	CTATATCAGGTGCTCCA
	6B	Lxyl COI F2C/T335-355	GTTTACCCCCCTTTATCAT*T*G
		Lxyl COI R2A/T421-451	AGTAATAAAATTAATTGCCCCTAAAATTG*T*G
	6C	Lluc COI F455-81	ATCATTAATATGCGATTAACTAATCTT
		Lluc COI R631-652	ATGTTGATAGAGAATTGGATCA

**Table 3:** List of probes developed for each simplex, duplex and triplex assay, as identified in Fig 1. The first 3–4 letters of probe names designate the taxa being targeted (except for FS1, which designates the marker), while “RC” means reverse complement. In probe sequences, bases preceded by a “+” sign are LNA bases.

Assay	Assay #	Probe name	Fluorophore	Probe sequence
*Duplex 1*	1A	Lalb COI 525–552	Cy5-	CAGCTTTCCTTCTACTTTTATCATTACC
	1B	AGM comp COI 636–649	FAM-LNA	C+AAT+C+CTTT+A+C+CAA
*Duplex 2*	2A	Ldaj COI RC 411–424	FAM-LNA	T+GAT+GAAA+T+A+C+CAG
	2B	Lda COI3P RC 788–797	Cy5-LNA	AG+C+C+CA+AA+CA
*Simplex 3*	3A	Ldd COI 531–560	FAM	TCCTTCTACTTTTATCTTTACCTGTTTTAG
*Duplex 4*	4A	FS1 AGM 62–86	FAM	ACTCAACATAAAGTATGCCAACTCG
	4B	FS1 EGM RC 21–47	Cy5	AGTACTGCTGTATACATTTTAAACGTC
*Duplex 5*	5A	Lmon COI 221–234	FAM-LNA	C+C+A+GATATAG+C+C+TT
	5B	Lfum COI 532–560	Cy5	TCTTTTACTCCTATCTTTACCCGTTTTAG
*Triplex 6*	6A	Lmat COI 116–139	Tex-615	AATACCATTGTTACAGCTCATGCT
	6B	Lxyl COI 381–412	Cy5	TAGATTTAGCTATTTTTTCCCTTCATTTAGCT
	6C	Lluc COI 532–561	FAM	TTTACTCCTTCTTTCTTTACCTGTTTTAGC

For initial singleplex testing, all probes were labelled with fluorescein (6-FAM) at the 5’ end and the quencher Iowa Black FQ (IBFQ) at the 3’ end. For subsequent duplex and triplex assay validation, probes labelled with the fluorophore Cy5 at the 5’ end and the quencher Iowa Black RQ (IBRQ) at the 3’ end and the fluorophore TEX-615 at the 5’ end and the quencher Iowa Black RQ (IBRQ) at the 3’ end were used. Additionally, for non-LNA probes (see section on LNA probes below), a ZEN^™^ (for 6-FAM probes) or TAO^™^ (for Cy5 probes) internal quencher was placed between the 9^th^ and 10^th^ base from the reporter dye on the 5’ end of the probe sequence. Those internal quenchers shorten the distance between dye and quencher and, in combination with the terminal 3’ quencher, provide a higher degree of quenching and lower initial background fluorescence. Duplex and triplex assays were analyzed for interactions between all primers and probes using Oligo Analyzer v1.2 and subsequently tested against the same panel of species used for the singleplex assays. Because the majority of the assays were designed in the COI-5P and COI-3P gene regions, care was taken to ensure that there was no overlap of amplicon regions in these assays.

#### Use of ARMS primers

Amplification Refractory Mutation System (ARMS) primers are useful when only one discriminatory SNP is available for the development of assays where discrimination is dependent on primers (i.e., probe is non-discriminatory). An artificial ARMS SNP can be added adjacent to an existing 3’ end SNP at either position 2 or position 3 of the primer to significantly increase the discriminatory ability of that primer [34]. ARMS primer combinations were tested against non-ARMS primer pairs to determine the combination that gave the best discrimination of non-target species while having a minimal effect on the specificity of the target species amplification.

#### Use of locked nucleic acid (LNA) probes

**Fig 1:**
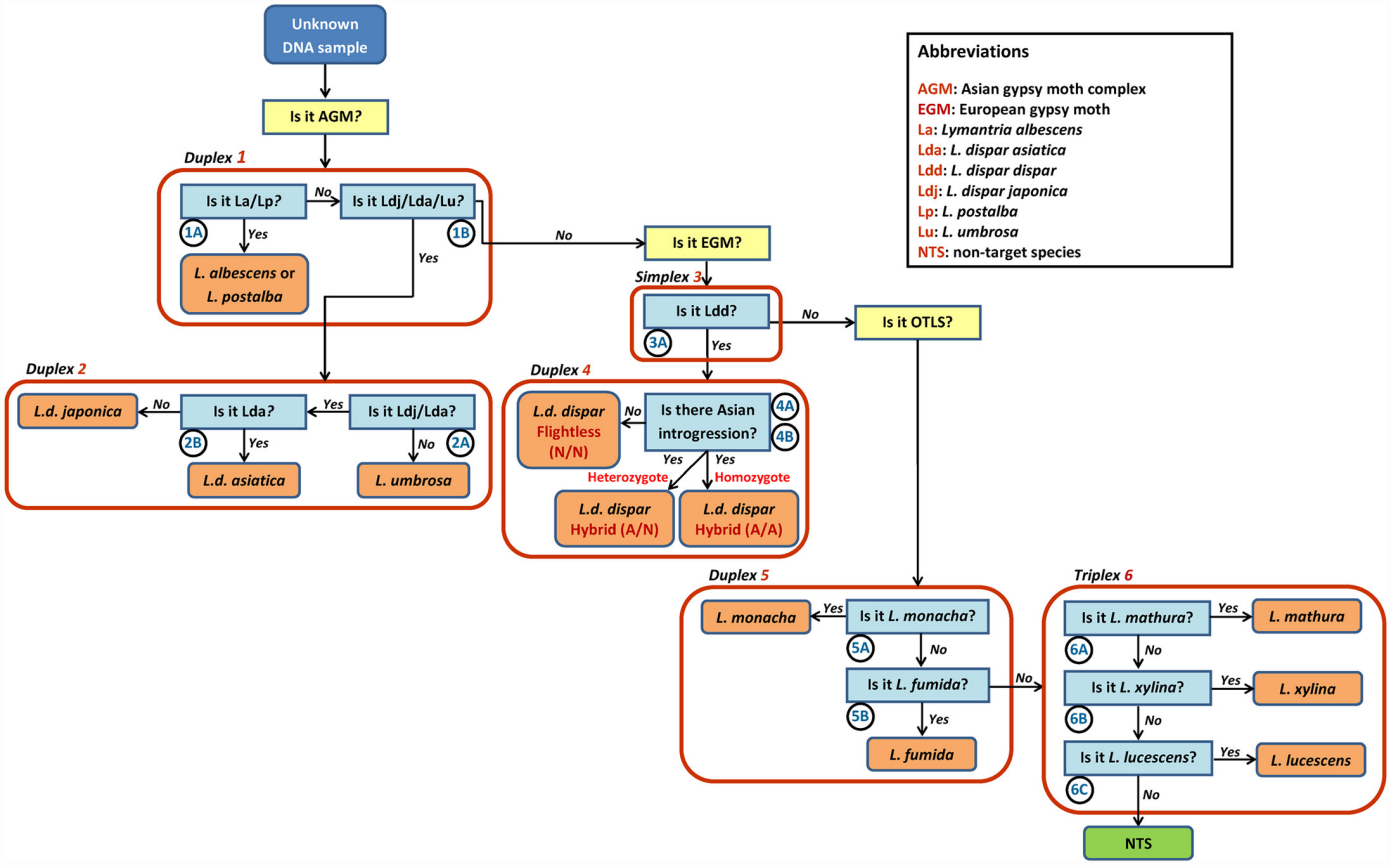
Architecture of AGM TaqMan assays: a “molecular key”. Flowchart depicting the parallel qPCR assays designed for the molecular identification of *Lymantria dispar* subspecies and eight additional *Lymantria* species, including those considered to be AGM. The assay scheme comprises three conceptual subgroups (yellow boxes), each encompassing a specific set of questions (light blue boxes): (i) Is the unknown a member of the AGM complex and, if so, which AGM species/subspecies is it? (ii) If the unknown is not AGM, is it EGM and, if so, does it show signs of Asian introgression? And (iii) if the unknown is neither AGM nor EGM, is it one of five other threatening *Lymantria* species (OTLS)? The assay is designed like a taxonomic key where molecular features substitute for morphological characters; it may therefore be thought of as a “molecular key”. Each unknown sample is processed in a sequential manner through the key, starting at the top left, until it can be assigned to a taxon or to the category “non-target species” (NTS; bottom right).

LNA nucleotides are used to increase the sensitivity and specificity of annealing in qPCR probes. A triplet of LNA nucleotides surrounding a single base mismatch site maximizes probe specificity [26]. LNA probes were used primarily in assays that were designed with single SNP discrimination (duplex 1A, duplex 2A, duplex 2B; Fig 1) and in assays where primers alone gave insufficient discrimination (duplex 5A; Fig 1). LNA probes were designed using IDT’s DNA Thermodynamics and Hybridization tool (biophysics.idtdna.com).

### SYBRGreen-based real-time PCR quantification for standardization of moth DNA concentration

DNA concentrations of all moth samples were standardized by qPCR quantification using lymantriine general primers (Table 2). Quantification and standardization of the DNA prior to its use in the TaqMan discrimination assays allowed us to confirm that DNA was present in a high enough concentration in all samples to ensure discrimination of all closely related species in the assays. It also simplified the interpretation and analysis of the TaqMan assay results. Lymantriine general primers were designed in a conserved region of the 5’ end of the COI gene using Oligo Explorer v1.2 and Oligo Analyzer v1.2. Degeneracy in the primers was kept to three degenerate bases per primer in an attempt to conserve the efficiency of the amplification reaction. The length of the amplicon was 128 bp.

Real-time PCR was performed with an Applied Biosystems 7500 Fast Real-Time PCR System (Life Technologies, Carlsbad, CA, USA). All reactions were performed in a final volume of 10 μL and contained 1x QuantiTect SYBR Green PCR Master Mix (Qiagen, Valencia, CA, USA), 0.5 μM of each of the lymantriine general primers (Table 2), and 1 μL of template DNA. Real-time PCR thermocycling conditions were set at 95°C for 15 min, followed by 40 cycles at 95°C for 15 s, 50°C for 30 s, and 65°C for 60 s. Fluorescence was read at the end of the extension step. Gene copy quantification was then performed using a Java program based on linear regression of efficiency [35], and sample DNA concentration was adjusted to 1000–2000 gene copies per μL (Ct value between 22 and 23), where possible. Ct value was determined by placing the fluorescence threshold (F_t_) at 5% of maximum fluorescence (F_max_).

### Validation

Specificity validation of all the assays was performed using the panel of specimens listed in Table 1. Real-time PCR amplification was conducted using 1x QuantiTect Multiplex PCR NoROX Master Mix, with 0.5 μM of each primer, 0.1 μM of TaqMan probe, and ~2,000 gene copies of template DNA, whenever possible, in a final reaction volume of 10 μL. Three technical replicates were performed for all reactions. Thermocycling conditions were set at 95°C for 15 min, followed by 45 cycles at 95°C for 15 s and 60°C for 90 s. Fluorescence was read at each cycle, at the end of the extension step. The fluorescence threshold (F_t_) was set at 10% of F_max_ for the analysis of these results to avoid false Ct values for any samples that may have a baseline drift.

Sensitivity of the TaqMan assays was evaluated in terms of both efficiency and limit of detection (LOD). For each target assay, experiments were conducted to (i) determine if Ct values were proportional to the amount of target template DNA (efficiency) and (ii) evaluate the LOD, which is the smallest amount of target DNA that can be detected for each of the assays. At least one isolate for each of the target species was selected, and TaqMan assay sensitivity was assessed on parallel sets of serial dilutions from the DNA stock.

To assess efficiency of the amplification reaction, TaqMan assays were run with serial dilutions of template DNA from the target species, with the DNA initially quantified using the COI-based lymantriine general primers described above. Standard curves were obtained by plotting the values of Ct against the log value of the target gene region copy number. Amplification reaction efficiency was calculated using the following formula:

$$E=\;\left(10^{\left(–1/slope\right)}–\;1\right)\;\times \;100$$

where *E* represents the amplification reaction efficiency and *slope* is the slope value of the line derived from the standard curve plot. Estimation of the LOD was done by performing 20 replicates of the TaqMan real-time PCR reactions for the lowest detectable DNA concentrations determined above. The lowest DNA concentration with a level of 95% successful amplification was identified as the LOD.

## Results

### Rationale for the choice of marker genes

In the course of developing the assay presented here, we compiled existing *Lymantria* marker sequences gleaned from public databases (GenBank, BOLD) and sequenced several other markers (both mitochondrial and nuclear) from the various species/subspecies for which we had samples (Table 1). Following thorough comparisons carried out for each set of marker sequences, it became apparent that the full COI gene (i.e., 5’ barcoding region [COI-5P] + remaining 3’ region [COI-3P]) contained enough polymorphism to allow separation of all targeted species and subspecies through the design of specific qPCR primers and probes. In addition, the large number of COI-5P sequences deposited in public databases considerably increased our confidence in sequence consistency and, as a result, in assay reliability. The only other marker we included is the “FS1” nuclear marker [20]. In the absence of markers diagnostic of female flight capability, we reasoned that FS1 could be used to determine whether insects identified as *L*. *dispar dispar*, on the basis of mitochondrial markers, are in fact AGM-EGM hybrids, some of which could have flight-capable females [22].

### Assay development and description

The architecture of the qPCR assays presented here resembles that of a standard taxonomic key, but one where genomic features substitute for morphological characters. The full assay is partitioned into three taxonomic subgroups (yellow shaded boxes; Fig 1): (i) AGM complex, (ii) EGM and (iii) other threatening lymantriine species (OTLS). Each subgroup comprises several independent assays that are run in simplex, duplex or triplex mode, for a total of two assay tubes per subgroup (red boxes; Fig 1) and six tubes for the whole assay when all reactions are run in parallel.

**Fig 2:**
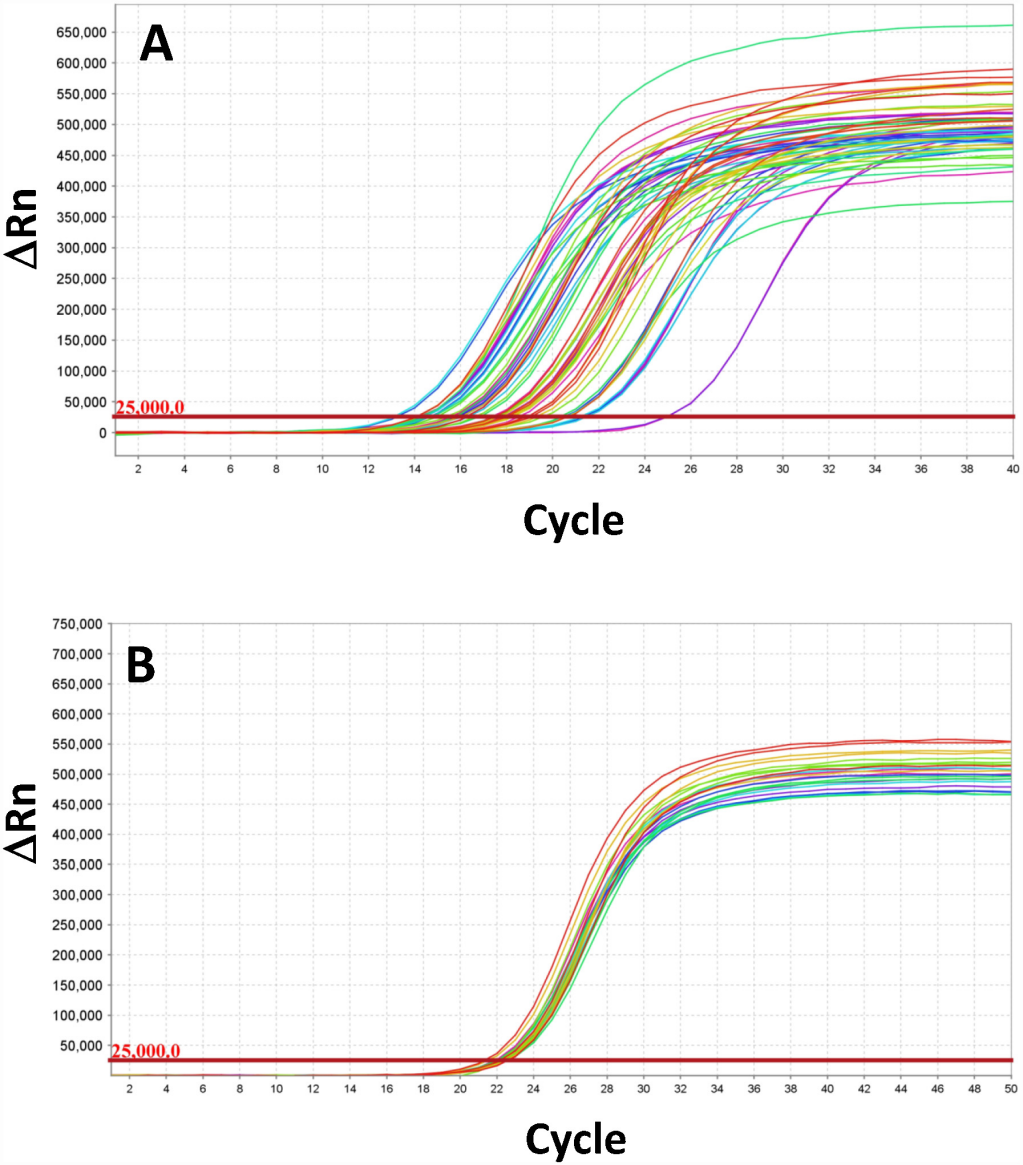
Amplification of undiluted and diluted (normalized) DNA samples. Comparison of amplification curves generated using COI lymantriine general primers (S1 File, tab 1) on multiple, undiluted samples **(A)**, and those obtained from the same samples following dilutions calculated to achieve the Ct of 22–23 **(B)**.

Each individual assay (light-blue shaded boxes; Fig 1) represents a dichotomic node featuring a question to which the qPCR run is expected to provide a “yes” or “no” answer, i.e. there should either be a clear amplification or no amplification at all. Of course, such a system will not perform adequately if provided “maybe” answers (e.g., a positive amplification but very late in the amplification cycle). To avoid this type of uncertainty, one must first select appropriate primers and probes that maximize target selectivity, but it is also critical to standardize the concentration of DNA used in each run. To this end, degenerate primers that amplify a small region of the COI-5P region in all species targeted by our assay (Table 1; see first tab of S1 File for details) were used as an indirect qPCR DNA quantification method. Low-Ct samples (i.e. highly concentrated) were then diluted to achieve a Ct of 22–23 (SYBR Green reading), using a calculation that assumes a doubling of DNA quantity at every amplification cycle. With the TaqMan probes employed in our assay, this dilution corresponds to a Ct of 25–28 for a mitochondrial marker and a Ct of 30–32 for a single-copy nuclear marker. Under these conditions, any run generating a Ct of >35 should be considered negative or may indicate DNA contamination. Fig 2 provides an example of how standardization of DNA concentration reduces variability in Ct values.

Below, we provide a description of each individual assay, including the rationale for SNP, primer and probe selection. Explanatory illustrations (sequence alignments and amplification curves) are provided herein for the first individual assay, but the reader is referred to S1 File for similar illustrations supporting the description of the other assays (one assay per tab). Primers and probes developed for each individual assay are presented in Tables 2 and 3.

#### Duplex Assay 1A: Is it *L*. *albescens*/*L*. *postalba*?

**Fig 3:**
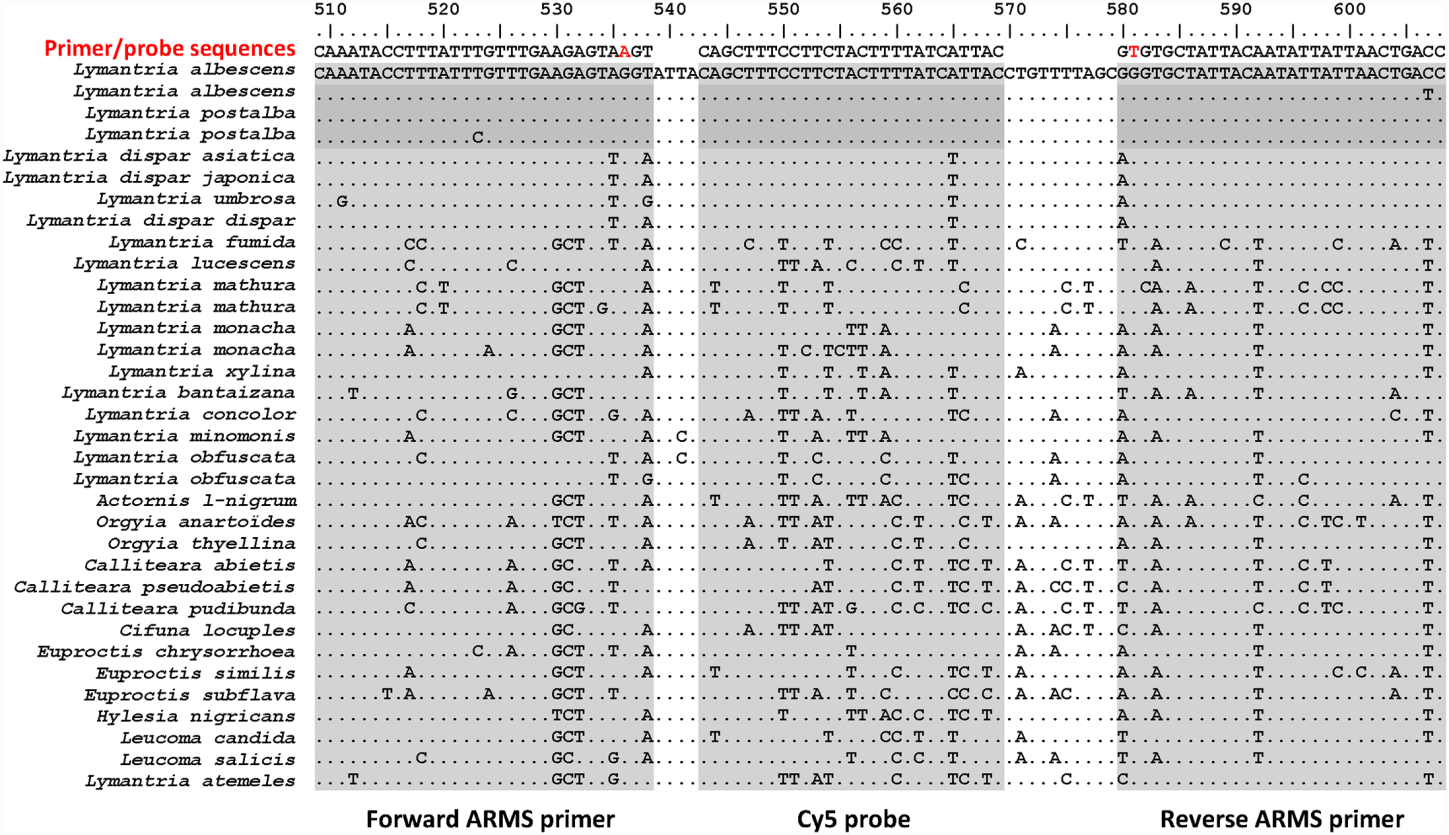
Example of the approach used to select and design qPCR primers and probes. Sequence alignment of the COI-5P region targeted for primer and probe design in the context of developing a qPCR assay that amplifies only *L*. *albescens* and *L*. *postalba* DNA. The primer and probe sequences are shown above the alignments. An ARMS base (red letter) was introduced into each primer to increase specificity. Sequences shown here were either gleaned from public databases or were obtained through specific PCR amplification followed by Sanger sequencing, as described in the Materials and methods section (see S1 File for details).

**Fig 4:**
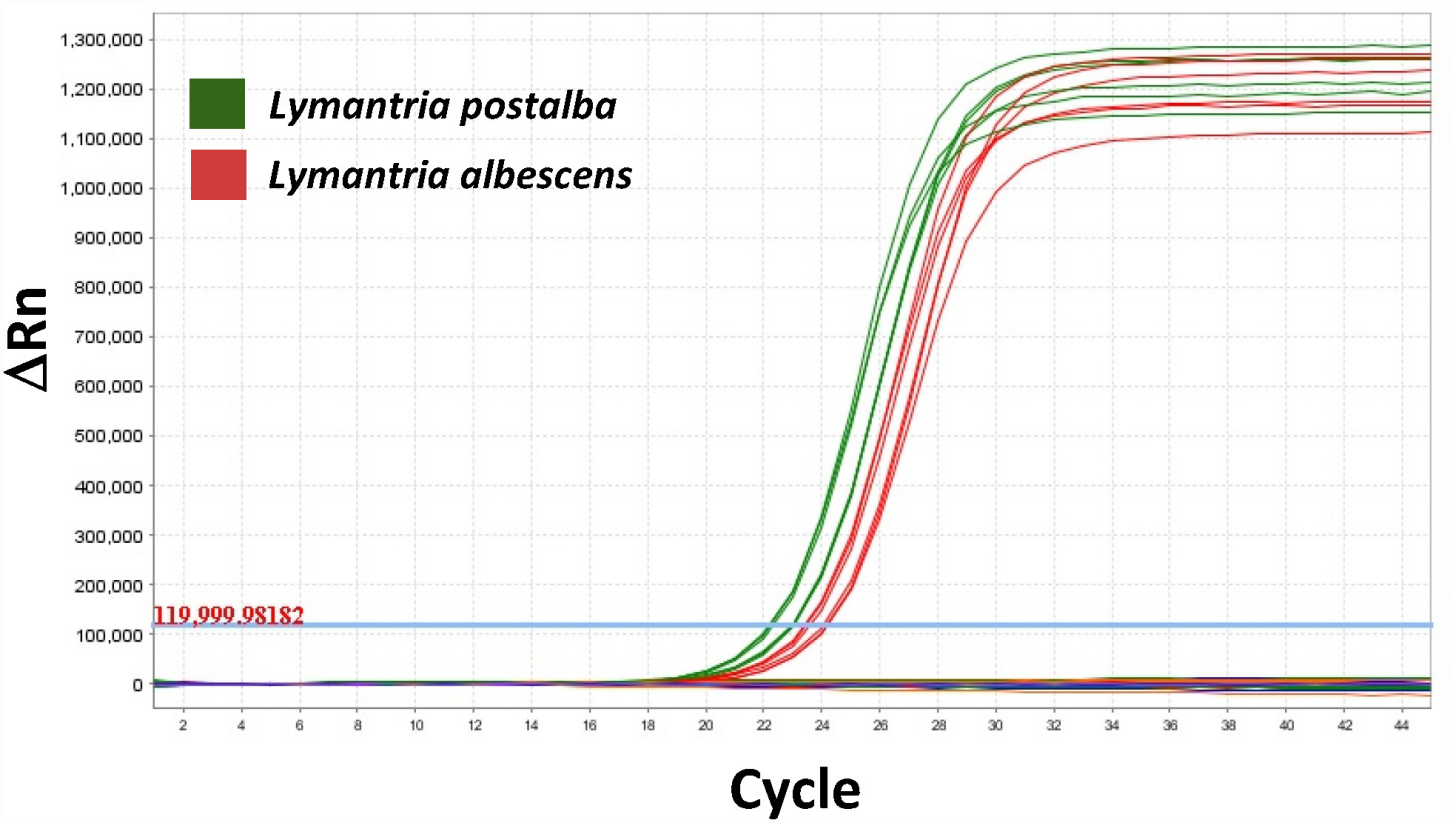
Example of a qPCR validation assay. Amplification curves obtained in validating the *L*. *albescens/L*. *postalba* assay tested on 61 samples (see S1 File, “Duplex assay 1A” tab, for details on samples). Only *L*. *albescens* (red traces) and *L*. *postalba* (green traces) samples generated positive amplifications (results of three technical replicates are shown for each sample).

This is the first of two assays designed to determine whether the unknown is a member of the AGM complex. It targets SNPs that are unique to the *L*. *albescens/L*. *postalba* species pair, which are here treated together because of the high degree of sequence identity displayed by their COI-5P regions. For this assay, discriminatory SNPs fall within the forward and reverse primer sequences; the Cy5 probe is non-discriminatory against *L*. *dispar dispar* and other members of the AGM complex (Fig 3). To enhance specificity, an ARMS base (red letter) was introduced into each primer. In validation tests, this assay produced amplifications for *L*. *albescens* and *L*. *postalba*, but none for any of the other species/subspecies examined (Fig 4 and S1 File).

#### Duplex Assay 1B: Is it *L*. *dispar asiatica/L*. *dispar japonica/L*. *umbrosa*?

This assay aims at detecting the presence of any of the three remaining members of the AGM complex in a single qPCR step. If neither assay 1A nor assay 1B produces a positive amplification, then the unknown is not AGM and the molecular key bifurcates to the EGM assay. Discriminatory SNPs for assay 1B fall within both the forward primer (except for discrimination against *L*. *dispar dispar*) and FAM-LNA probe, which are near the 3’ end of the COI-5P region; the reverse primer is located within the COI-3P region, where SNPs enable discrimination against *L*. *albescens/L*. *postalba* (S1 File). If a positive amplification is obtained in assay 1B, then the chart points towards additional assays that will determine which of the three targeted AGM complex members is present in the sample.

#### Duplex Assay 2A: Is it *L*. *dispar asiatica/L*. *dispar japonica*?

This assay provides discrimination between *L*. *umbrosa* and the two Asian *L*. *dispar* subspecies: a negative amplification identifies the unknown as *L*. *umbrosa* (Fig 1). Both primers and probe fall within the COI-5P region, and discrimination against *L*. *umbrosa* is provided by a T/A substitution located in the FAM-LNA probe region (S1 File).

#### Duplex Assay 2B: Is it *L*. *dispar asiatica*?

If the previous assay indicated that the unknown is either *L*. *dispar asiatica* or *L*. *dispar japonica*, assay 2B will generate amplification only if the sample is *L*. *dispar asiatica*; absence of amplification implies that the unknown is *L*. *dispar japonica*. Discrimination is here provided by a G/A substitution within the region targeted by the Cy5-LNA probe, which is located in the 3’ portion of COI (S1 File).

#### Simplex Assay 3A: Is it *L*. *dispar dispar*?

When the above-described duplex assay 1 generates negative results for the AGM complex, the molecular key then tests the hypothesis that the unknown is EGM. To this end, discrimination between *L*. *dispar dispar* and other lymnatriids is provided by a COI-5P-based FAM probe that is specific to EGM. Degeneracy is introduced at one site in the forward primer to take into account some variation observed among independent *L*. *dispar dispar* COI-5P sequences. It must be pointed out here that the design of this assay does not take into account any AGM complex sequences, as this possibility is already eliminated at this intersection of the molecular key (S1 File).

#### Duplex Assays 4A and 4B: Does *L*. *dispar dispar* show evidence of Asian introgression?

**Fig 5:**
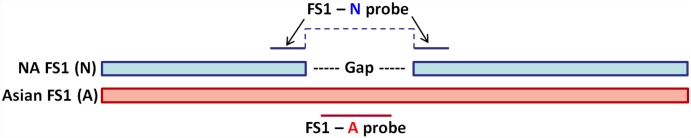
Diagram illustrating the principle guiding the design of qPCR probes specific to the North American (N) and Asian (A) alleles of the FS1 nuclear marker [20]. See Results section and S1 File for details.

**Fig 6:**
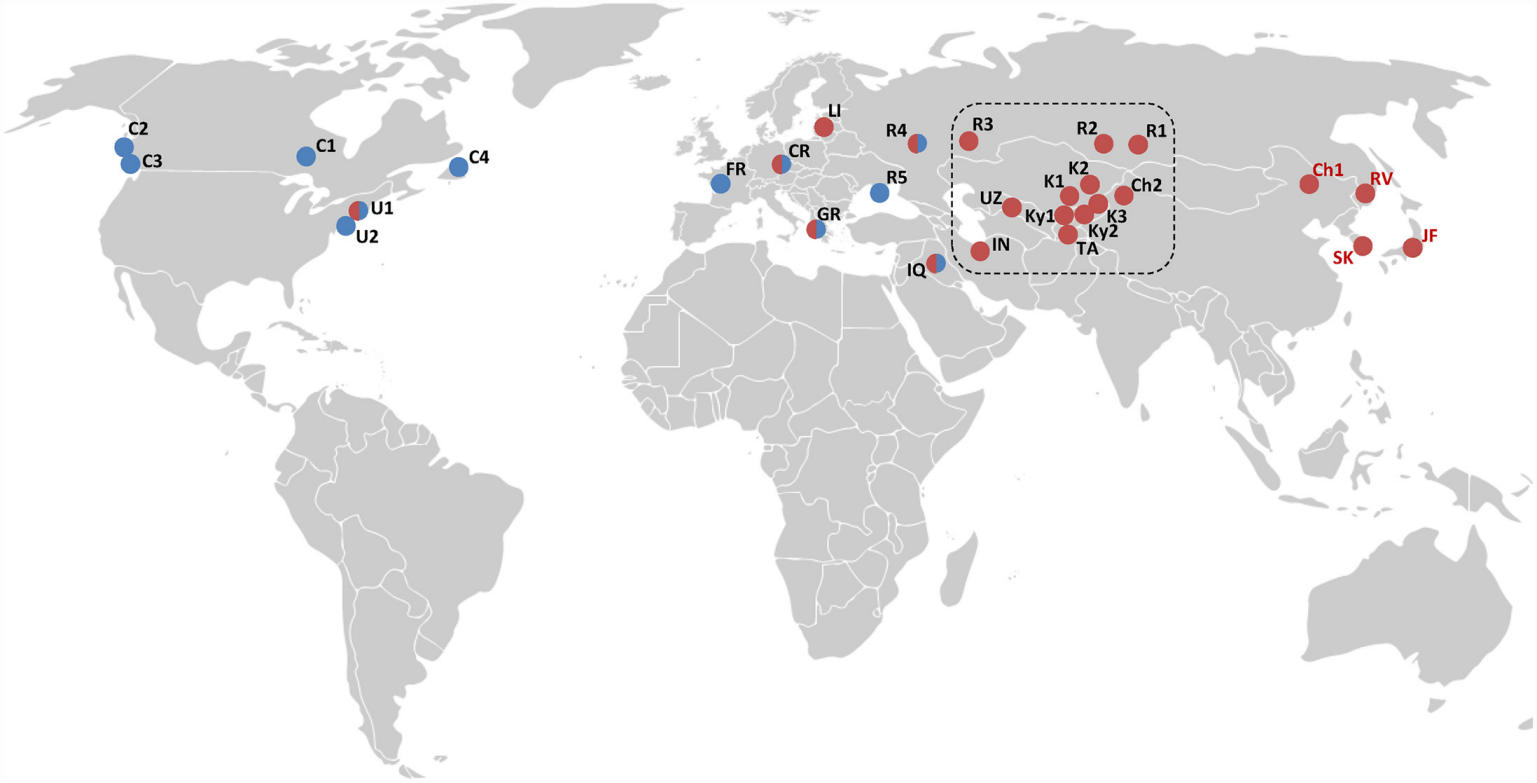
Geographical distribution of FS1 genotypes, as determined using *Duplex assay 4*, for gypsy moths identified as *L*. *dispar dispar* using *Simplex assay 3*. Blue circles: homozygous for the FS1-N allele; red circles: homozygous for the FS1-A allele; blue/red circles: heterozygous for the N and A alleles. Black letters near each circle identify specimens identified as *L*. *dispar dispar* using *Simplex assay 3*; red letters designate *L*. *dispar asiatica* and *L*. *dispar japonica* positive controls (refer to Table 4 for details). Box: central Asia region rich in specimens that are homozygous for the FS1-A allele while displaying *L*. *dispar dispar* COI barcode. Background map is from https://commons.wikimedia.org/wiki/.

Previous studies have reported the existence of gypsy moth populations, primarily from central Asia, that feature mitochondrial DNA sequences diagnostic of *L*. *dispar dispar* while displaying biological characteristics that are typical of AGM, including flight capability in females [22]. From a regulatory standpoint, such insects may need to be treated as AGM. To assess the occurrence of Asian introgression into EGM, we made use of the FS1 nuclear marker, for which North American (“N”) and Asian (“A”) alleles have been described and where the latter features a 103 bp insertion relative to the former [20]. Thus, the present assay is not a typical SNP-based assay; rather, discrimination between the two alleles relies on the design of two separate probes: a Cy5 probe that spans the gap in the North American allele and a FAM probe that is specific to the Asian insertion (Fig 5). Specificity of the N allele probe is further enhanced by the presence of two substitutions within the region targeted by the probe (S1 File). Validation of this assay involved the testing of *bona fide* Asian gypsy moths (*L*. *dispar asiatica* and *L*. *dispar japonica*), and *L*. *dispar dispar* specimens, as well as that of several other moths suspected of being hybrids, either based on earlier reports or on their geographical origins (i.e., near the boundary of the *L*. *dispar dispar* and *L*. *dispar asiatica* ranges). As expected, the *L*. *dispar asiatica* and *L*. *dispar japonica* specimens examined (from eastern China, South Korea, Russian Far East and Japan) were homozygous for the A allele while most *L*. *dispar dispar* samples from North America were homozygous for the N allele. However, specimens from Siberia and Lithuania, previously reported to have flight-capable females while displaying *L*. *dispar dispar* COI-5P sequences [22], were homozygous for the A allele, as were several other specimens from central Asia, including Kazakhstan, Kyrgyzstan, Tajikistan, and Iran (Table 4; Fig 6). Specimens from the Czech Republic and Greece were heterozygous for the FS1 marker, as was a specimen from Connecticut (heterozygosity of some specimens from Connecticut was noted earlier [22]). On the European continent, specimens from France and from the Crimean Peninsula were homozygous for the N allele (Table 4; Fig 6). Regulatory implications of these findings are addressed in the Discussion section.

**Table 4:** FS1 genotyping (*Duplex assay 4*) for 25 gypsy moth specimens identified as *L*. *dispar dispar* using *Simplex assay 3* (Fig 1).

					Ct for FS1 –N		Ct for FS1 –A		
Sample id	COI-based id	Country	Region	Code	Run 1^2^	Run 2	Run 1	Run 2	Genotype^3^
CFS-6	***L*. *d*. *asiatica***^1^	China	Heilongjang	Ch1	-	-	29.11	29.07	**AA**
AGM-25	***L*. *d*. *asiatica***	Russia	Vladivostok	RV	-	-	32.04	32.29	**AA**
AGM-26	***L*. *d*. *asiatica***	South Korea	Kangwon	SK	-	-	31.97	32.20	**AA**
AGM-31	***L*. *d*. *japonica***	Japan	Fujikawa	JF	-	-	30.90	30.93	**AA**
LEP0195	*L*. *d*. *dispar*	China	Xingjiang	Ch2	-	-	31.86	31.41	**AA**
CFS-8	*L*. *d*. *dispar*	Russia	Siberia	R1	-	-	28.23	28.83	**AA**
LEP0299	*L*. *d*. *dispar*	Russia	Western Siberia	R2	-	-	32.02	31.96	**AA**
LEP0416	*L*. *d*. *dispar*	Russia	Bashkortostan	R3	-	-	32.85	32.98	**AA**
LEP0479	*L*. *d*. *dispar*	Kyrgyzstan	Toktogul	Ky1	-	-	29.04	29.34	**AA**
LEP0476	*L*. *d*. *dispar*	Kyrgyzstan	Ala-Too	Ky2	-	-	31.44	30.73	**AA**
LEP0151	*L*. *d*. *dispar*	Tajikistan	Kondara	TA	-	-	31.91	32.07	**AA**
LEP0295	*L*. *d*. *dispar*	Uzbekistan	Chimgan	UZ	-	-	32.00	32.14	**AA**
LEP0456	*L*. *d*. *dispar*	Kazakhstan	Koram	K1	-	-	29.72	29.56	**AA**
LEP0470	*L*. *d*. *dispar*	Kazakhstan	Usek	K2	-	-	31.03	31.50	**AA**
LEP0469	*L*. *d*. *dispar*	Kazakhstan	Chingilsu Valley	K3	-	-	29.97	30.28	**AA**
CFS-10	*L*. *d*. *dispar*	Lithuania	Unavailable	LI	-	-	28.49	28.41	**AA**
LEP0146	*L*. *d*. *dispar*	Iran	Kuh-e-Sendan	IN	-	-	34.65	34.74	**AA**
LEP0465	*L*. *d*. *dispar*	Russia	Nizhny Novgorod	R4	29.88	29.76	30.22	30.55	**NA**
LEP0147	*L*. *d*. *dispar*	Iraq	Kirkuk	IQ	32.12	32.26	33.34	33.36	**NA**
CFS-11	*L*. *d*. *dispar*	Greece	Unavailable	GR	29.30	29.30	29.22	29.00	**NA**
LEP0492	*L*. *d*. *dispar*	Czech Republic	Lulec	CR	31.98	31.85	30.93	30.84	**NA**
CFS-12	*L*. *d*. *dispar*	USA	CT	U1	28.95	29.04	30.17	29.93	**NA**
LEP0427	*L*. *d*. *dispar*	Russia	Crimea	R5	28.20	28.29	-	-	**NN**
LEP0234	*L*. *d*. *dispar*	France	Touraine	FR	31.38	31.26	-	-	**NN**
AGM-23	*L*. *d*. *dispar*	USA	NJ (Newark)	U2	29.21	29.28	-	-	**NN**
AGM-22	*L*. *d*. *dispar*	Canada	ON	C1	27.18	26.94	-	-	**NN**
AGM162	*L*. *d*. *dispar*	Canada	BC	C2	26.55	26.75	-	-	**NN**
LEP0035	*L*. *d*. *dispar*	Canada	BC	C3	31.04	31.34	-	-	**NN**
LEP0037	*L*. *d*. *dispar*	Canada	NF	C4	30.76	30.87	-	-	**NN**

^1^ First four samples: positive controls for the AA genotype.

^2^ “ -” indicates absence of amplification.

^3^ AA: homozygous for the Asian FS1 allele; NN: homozygous for the North America FS1 allele; NA: heterozygous.

The duplex assay 5 and triplex assay 6 (Fig 1) described below may be regarded as forming a single multiplex assay where each individual assay need not be treated in a sequential fashion; separation of these two assays and the sequential presentation of individual assays are done for convenience only.

#### Duplex Assay 5A: Is it *L*. *monacha*?

In cases where assay 3A (Is it *L*. *dispar dispar*?) produces no amplification, the dichotomic key redirects the identification process to the OTLS subgroup, where the first assay targets *L*. *monacha* (Fig 1). Here, discrimination is provided primarily by the FAM-LNA probe and the reverse primer, both of which fall within COI-5P regions that feature several SNPs relative to other lymantriines (S1 File).

#### Duplex Assay 5B: Is it *L*. *fumida*?

For the *L*. *fumida* assay, both primers and the Cy5 probe contribute to discriminating this species from others. Targeted regions are within the COI-5P marker and contain many SNPs (S1 File).

#### Triplex Assays 6A, 6B and 6C: Is it *L*. *mathura*, *L*. *xylina or L*. *lucescens*?

Negative amplification at the previous step brings the identification process to this last set of assays, which are run in triplex for convenience. All three individual assays are similar to the *L*. *fumida* assay in that, for each, discrimination is provided by both primers and the probe (Tables 3 and 4). In the *L*. *mathura* assay, degeneracy is introduced at two sites in the forward primer to account for some sequence variation among populations of different geographic origins. In the *L*. *xylina* assay, ARMS bases are introduced in both forward and reverse primers to enhance discrimination relative to the non-target species (S1 File). In cases where upstream assays have not enabled identification of the unknown, failure to obtain an amplification in this triplex assay leads to the conclusion that the unknown may not be assigned to any of the species or subspecies targeted by the present molecular identification key (Fig 1).

To assist users in the identification process, we provide an Excel sheet tool where results of each individual assay (*yes* or *no* amplification) may be entered for automatic species/subspecies assignment after the full suite of assays has been run (S2 File).

### Assay specificity validation

To assess the specificity of our molecular key, each individual assay was tested using a set of species/subspecies considered pertinent to the assay being evaluated. A total of 105 specimens were used for validation purposes (Table 5), a list that includes specimens processed to generate the FS1 assay data presented in Table 4, plus three additional specimens that were used for FS1 genotyping only (samples CFIA-LEP0146, CFIA-LEP0147 and AGM-0022 in Table 4). Details of each validation test may be found in S1 File, a summary of which is presented in Table 5.

**Table 5:** Specificity validation results for the different TaqMan assays.

Specimen ID	Species/subspecies	Expected positive result^1^	Result per assay^2^											
			1A	1B	2A	2B	3A	4A	4B	5A	5B	6A	6B	6C
**AGM complex**														
CFIA-LEP0246	*Lymantria albescens*	1A	+	-	•	•	•	•	•	•	•	•	•	•
CFIA-LEP0247	*Lymantria albescens*	1A	+	-	•	•	•	•	•	•	•	•	•	•
CFIA-LEP0306	*Lymantria albescens*	1A	+	-	•	•	•	•	•	•	•	•	•	•
CFIA-LEP0307	*Lymantria albescens*	1A	+	-	•	•	•	•	•	•	•	•	•	•
AGM-0537	*Lymantria albescens*	1A	+	-	•	•	•	•	•	•	•	•	•	•
AGM-0538	*Lymantria albescens*	1A	+	-	•	•	•	•	•	•	•	•	•	•
AGM-0545	*Lymantria albescens*	1A	+	-	•	•	•	•	•	•	•	•	•	•
AGM-0565	*Lymantria albescens*	1A	+	-	•	•	•	•	•	•	•	•	•	•
AGM-0566	*Lymantria albescens*	1A	+	-	•	•	•	•	•	•	•	•	•	•
AGM-0581	*Lymantria albescens*	1A	+	-	•	•	•	•	•	•	•	•	•	•
AGM-0582	*Lymantria albescens*	1A	+	-	•	•	•	•	•	•	•	•	•	•
AGM-0587	*Lymantria albescens*	1A	+	-	•	•	•	•	•	•	•	•	•	•
AGM-0588	*Lymantria albescens*	1A	+	-	•	•	•	•	•	•	•	•	•	•
AGM-0603	*Lymantria albescens*	1A	+	-	•	•	•	•	•	•	•	•	•	•
AGM-0604	*Lymantria albescens*	1A	+	-	•	•	•	•	•	•	•	•	•	•
AGM-0606	*Lymantria albescens*	1A	+	-	•	•	•	•	•	•	•	•	•	•
AGM-0607	*Lymantria albescens*	1A	+	-	•	•	•	•	•	•	•	•	•	•
AGM-0500	*Lymantria postalba*	1A	+	-	•	•	•	•	•	•	•	•	•	•
AGM-0501	*Lymantria postalba*	1A	+	-	•	•	•	•	•	•	•	•	•	•
AGM-0502	*Lymantria postalba*	1A	+	-	•	•	•	•	•	•	•	•	•	•
AGM-0514	*Lymantria postalba*	1A	+	-	•	•	•	•	•	•	•	•	•	•
AGM-0515	*Lymantria postalba*	1A	+	-	•	•	•	•	•	•	•	•	•	•
AGM-0520	*Lymantria postalba*	1A	+	-	•	•	•	•	•	•	•	•	•	•
AGM-0521	*Lymantria postalba*	1A	+	-	•	•	•	•	•	•	•	•	•	•
AGM-0526	*Lymantria postalba*	1A	+	-	•	•	•	•	•	•	•	•	•	•
AGM-0527	*Lymantria postalba*	1A	+	-	•	•	•	•	•	•	•	•	•	•
AGM-0529	*Lymantria postalba*	1A	+	-	•	•	•	•	•	•	•	•	•	•
AGM-0530	*Lymantria postalba*	1A	+	-	•	•	•	•	•	•	•	•	•	•
AGM-0009	*Lymantria dispar asiatica*	1B; 2A; 2B	-	+	+	+	•	•	•	•	•	•	•	•
AGM-0018	*Lymantria dispar asiatica*	1B; 2A; 2B	-	+	+	+	•	•	•	•	•	•	•	•
AGM-0024	*Lymantria dispar asiatica*	1B; 2A; 2B	-	+	+	+	•	•	•	•	•	•	•	•
AGM-0026	*Lymantria dispar asiatica*	1B; 2A; 2B	-	+	+	+	•	**+**^3^	**-**	•	•	•	•	•
AGM-0025	*Lymantria dispar asiatica*	1B; 2A; 2B	-	+	+	+	•	**+**^3^	**-**	•	•	•	•	•
AGM-0027	*Lymantria dispar asiatica*	1B; 2A; 2B	-	+	+	+	•	•	•	•	•	•	•	•
CFS-0003	*Lymantria dispar asiatica*	1B; 2A; 2B	-	+	+	+	•	•	•	•	•	•	•	•
CFS-0004	*Lymantria dispar asiatica*	1B; 2A; 2B	-	+	+	+	•	•	•	•	•	•	•	•
CFS-0005	*Lymantria dispar asiatica*	1B; 2A; 2B	-	+	+	+	•	•	•	•	•	•	•	•
CFS-0006	*Lymantria dispar asiatica*	1B; 2A; 2B	-	+	+	+	•	**+**^3^	**-**	•	•	•	•	•
CFS-0007	*Lymantria dispar asiatica*	1B; 2A; 2B	-	+	+	+	•	•	•	•	•	•	•	•
CFS-0009	*Lymantria dispar asiatica*	1B; 2A; 2B	-	+	+	+	•	•	•	•	•	•	•	•
AGM-0001	*Lymantria dispar japonica*	1B; 2A	-	+	+	-	•	•	•	•	•	•	•	•
AGM-0015	*Lymantria dispar japonica*	1B; 2A	-	+	+	-	•	•	•	•	•	•	•	•
AGM-0016	*Lymantria dispar japonica*	1B; 2A	-	+	+	-	•	•	•	•	•	•	•	•
AGM-0017	*Lymantria dispar japonica*	1B; 2A	-	+	+	-	•	•	•	•	•	•	•	•
AGM-0031	*Lymantria dispar japonica*	1B; 2A	-	+	+	-	•	**+**^3^	**-**	•	•	•	•	•
AGM-0033	*Lymantria dispar japonica*	1B; 2A	-	+	+	-	•	•	•	•	•	•	•	•
AGM-0034	*Lymantria dispar japonica*	1B; 2A	-	+	+	-	•	•	•	•	•	•	•	•
CFS-0001	*Lymantria dispar japonica*	1B; 2A	-	+	+	-	•	•	•	•	•	•	•	•
CFIA-LEP0450	*Lymantria dispar japonica*	1B; 2A	-	+	+	-	•	•	•	•	•	•	•	•
CFIA-LEP0451	*Lymantria dispar japonica*	1B; 2A	-	+	+	-	•	•	•	•	•	•	•	•
CFIA-LEP0453	*Lymantria dispar japonica*	1B; 2A	-	+	+	-	•	•	•	•	•	•	•	•
CFIA-LEP0452	*Lymantria umbrosa*	1B	-	+	-	•	•	•	•	•	•	•	•	•
**EGM and other *L*. *dispar* specimens of uncertain subspecies designation**^4^														
CFIA-LEP0035	*Lymantria dispar dispar*	3A; 4A or 4B	-	-	•	•	+	-	+	•	•	•	•	•
CFIA-LEP0037	*Lymantria dispar dispar*	3A; 4A or 4B	-	-	•	•	+	-	+	•	•	•	•	•
CFIA-LEP0145	*Lymantria dispar*	3A; 4A or 4B	-	-	•	•	+	+^5^	-	•	•	•	•	•
CFIA-LEP0151	*Lymantria dispar*	3A; 4A or 4B	-	-	•	•	+	+	-	•	•	•	•	•
CFIA-LEP0195	*Lymantria dispar*	3A; 4A or 4B	-	-	•	•	+	+	-	•	•	•	•	•
CFIA-LEP0234	*Lymantria dispar dispar*	3A; 4A or 4B	-	-	•	•	+	-	+	•	•	•	•	•
CFIA-LEP0295	*Lymantria dispar*	3A; 4A or 4B	-	-	•	•	+	+	-	•	•	•	•	•
CFIA-LEP0299	*Lymantria dispar*	3A; 4A or 4B	-	-	•	•	+	+	-	•	•	•	•	•
CFIA-LEP0416	*Lymantria dispar*	3A; 4A or 4B	-	-	•	•	+	+	-	•	•	•	•	•
CFIA-LEP0427	*Lymantria dispar dispar*	3A; 4A or 4B	-	-	•	•	+	-	+	•	•	•	•	•
CFIA-LEP0456	*Lymantria dispar*	3A; 4A or 4B	-	-	•	•	+	+	-	•	•	•	•	•
CFIA-LEP0465	*Lymantria dispar*	3A; 4A or 4B	-	-	•	•	+	+	+	•	•	•	•	•
CFIA-LEP0469	*Lymantria dispar*	3A; 4A or 4B	-	-	•	•	+	+	-	•	•	•	•	•
CFIA-LEP0470	*Lymantria dispar*	3A; 4A or 4B	-	-	•	•	+	+	-	•	•	•	•	•
CFIA-LEP0476	*Lymantria dispar*	3A; 4A or 4B	-	-	•	•	+	+	-	•	•	•	•	•
CFIA-LEP0479	*Lymantria dispar*	3A; 4A or 4B	-	-	•	•	+	+	-	•	•	•	•	•
CFIA-LEP0492	*Lymantria dispar dispar*	3A; 4A or 4B	-	-	•	•	+	+	+	•	•	•	•	•
AGM-0023	*Lymantria dispar dispar*	3A; 4A or 4B	-	-	•	•	+	-	+	•	•	•	•	•
AGM-0151	*Lymantria dispar dispar*	3A; 4A or 4B	-	-	•	•	+	-	+	•	•	•	•	•
AGM-0162	*Lymantria dispar dispar*	3A; 4A or 4B	-	-	•	•	+	-	+	•	•	•	•	•
CFS-0008	*Lymantria dispar*	3A; 4A or 4B	-	-	•	•	+	+	-	•	•	•	•	•
CFS-0010	*Lymantria dispar*	3A; 4A or 4B	-	-	•	•	+	+	-	•	•	•	•	•
CFS-0011	*Lymantria dispar dispar*	3A; 4A or 4B	-	-	•	•	+	+	+	•	•	•	•	•
CFS-0012	*Lymantria dispar dispar*	3A; 4A or 4B	-	-	•	•	+	+	+	•	•	•	•	•
**OTLS**														
AGM-0036	*Lymantria monacha*	5A	-	-	•	•	-	•	•	+	-	-	-	-
AGM-0067	*Lymantria monacha*	5A	-	-	•	•	-	•	•	+	-	-	-	-
CFS-0014	*Lymantria monacha*	5A	-	-	•	•	-	•	•	+	-	-	-	-
CFIA-LEP0341	*Lymantria monacha*	5A	-	-	•	•	-	•	•	+	-	-	-	-
AGM-0047	*Lymantria fumida*	5B	-	-	•	•	-	•	•	-	+	-	-	-
AGM-0002	*Lymantria mathura*	6A	-	-	•	•	-	•	•	-	-	+	-	-
AGM-0050	*Lymantria mathura*	6A	-	-	•	•	-	•	•	-	-	+	-	-
AGM-0051	*Lymantria mathura*	6A	-	-	•	•	-	•	•	-	-	+	-	-
AGM-0052	*Lymantria mathura*	6A	-	-	•	•	-	•	•	-	-	+	-	-
AGM-0054	*Lymantria mathura*	6A	-	-	•	•	-	•	•	-	-	+	-	-
CFIA-LEP0253	*Lymantria xylina*	6B	-	-	•	•	-	•	•	-	-	-	+	-
CFIA-LEP0311	*Lymantria xylina*	6B	-	-	•	•	-	•	•	-	-	-	+	-
AGM-0068	*Lymantria lucescens*	6C	-	-	•	•	-	•	•	-	-	-	-	+
CFIA-LEP0093	*Lymantria lucescens*	6C	-	-	•	•	-	•	•	-	-	-	-	+
CFIA-LEP0094	*Lymantria lucescens*	6C	-	-	•	•	-	•	•	-	-	-	-	+
**Related species**														
AGM-0170	*Lymantria atemeles*	-	-	-	•	•	-	•	•	-	-	-	-	-
AGM-0071	*Lymantria bantaizana*	-	-	-	•	•	-	•	•	-	-	-	-	-
AGM-0164	*Lymantria concolor*	-	-	-	•	•	-	•	•	-	-	-	-	-
AGM-0040	*Lymantria minimonis*	-	-	-	•	•	-	•	•	-	-	-	-	-
AGM-0134	*Orgyia thyellina*	-	-	-	•	•	-	•	•	-	-	-	-	-
AGM-0093	*Leucomasalicis*	-	-	-	•	•	-	•	•	-	-	-	-	-
AGM-0096	*Leucoma salicis*	-	-	-	•	•	-	•	•	-	-	-	-	-
AGM-0138	*Arctornis I-nigrum*	-	-	-	•	•	-	•	•	-	-	-	-	-
AGM-0139	*Arctornis I-nigrum*	-	-	-	•	•	-	•	•	-	-	-	-	-
AGM-0107	*Calliteara abietis*	-	-	-	•	•	-	•	•	-	-	-	-	-
AGM-0115	*Calliteara pudibunda*	-	-	-	•	•	-	•	•	-	-	-	-	-
AGM-0127	*Cifuna locuples*	-	-	-	•	•	-	•	•	-	-	-	-	-
AGM-0132	*Cifuna locuples*	-	-	-	•	•	-	•	•	-	-	-	-	-
AGM-0121	*Euproctis similis*	-	-	-	•	•	-	•	•	-	-	-	-	-

^1^ Assays identified in Results section

^2^ “+”: amplification at expected Ct; “ -”: no amplification; “•”: not tested

^3^ Positive controls for the Asian FS1 allele

^4^ All specimens found in this section generated a positive amplification in the EGM assay (i.e., *Simplex assay 3*)

^5^ This amplification had a Ct of ~39, above the threshold of Ct = 35 set for this assay.

Notwithstanding the *L*. *dispar* specimens whose subspecies status was considered uncertain, all identifications provided by the molecular assays matched those made by taxonomists before DNA extraction. Most unspecified *L*. *dispar* specimens that we tested were from central Asia; while all of them were identified as *L*. *dispar dispar* using the mtDNA-based assay (simplex assay 3), they were all homozygous for the Asian FS1 allele (Fig 6), suggesting that these insects have an *L*. *dispar asiatica* genetic background. One specimen from Lebanon (CFIA-LEP0145; Table 5) generated a late amplification (Ct ~39) for the Asian FS1 allele, suggesting the presence of contaminants or substitution(s) in the region(s) targeted by the FS1 primers and/or probes.

### Assay sensitivity and direct PCR

All six assays developed here displayed a level of qPCR efficiency close to 100% and all showed a very high degree of sensitivity, with LOD values ≤ 25 COI copies (Table 6).

**Table 6:** Sensitivity: efficiency/limit of detection results.

Assay	Assay #	% Efficiency	LOD (COI copies/μL)
*Duplex 1*	1A	113.3	25
	1B	103.7	3
*Duplex 2*	2A	100.7	9
	2B	105.6	5
*Simplex 3*	3A	95.6	2
*Duplex 4*	4A	94.3	4
	4B	97.4	4
*Duplex 5*	5A	105.1	5
	5B	124.0	2
*Triplex 6*	6A	98.1	5
	6B	94.8	17
	6C	98.0	10

Given that simplification of the DNA extraction step could further reduce the time required to run the present set of assays, we examined the possibility of using a “direct PCR” approach, where the assay is run on an egg homogenate (see Materials and methods for details), as opposed to a purified DNA extract. As we were not able to obtain fresh eggs for most species and subspecies considered here, we ran our tests on *L*. *dispar dispar* eggs only. Homogenates of 2 and 4 eggs contained ~250,000 and ~500,000 COI gene copies, respectively. A 100-200x dilution of such homogenates is suitable for running the TaqMan assays.

## Discussion

The present suite of TaqMan assays was developed in response to a need expressed by the CFIA, the federal agency that has the responsibility of identifying potential FIAS intercepted at Canadian ports, including AGM and other related lymantriines considered a threat to North American forest resources. In developing the assays, we focused on four features for which improvements were needed relative to existing molecular assays: (i) accuracy/reliability of identification, (ii) resolution at the subspecies or population level, (iii) scope (i.e., number of species the assay can identify), and (iv) rapidity. Clearly, the method proposed here achieves significant improvements relative to earlier AGM assays, particularly when all four features are considered together.

The CFIA’s diagnostic entomology lab has so far relied primarily on the well known “NB” assay [19,22] for AGM identification, along with a second, microsatellite-based assay [22,36]. The NB system is a method that involves the digestion of COI-5P PCR amplicons with two restriction enzymes, each targeting SNPs that allow identification of unknown samples as North American, European/Siberian or Asian. Although this assay has often proven very useful, cases of ambiguous identification have been encountered [16,22], and it is suboptimal in regard to the other three features for which we sought improvements. Similarly, the microsatellite assay has been shown to provide limited subspecies resolution [20]. In addition, sample processing using these two assays takes two full days while the method we propose here takes less than a day (rapidity can be further improved by the direct PCR approach we developed). Thus, our suite of TaqMan assays has clear potential to enhance AGM diagnostic capacity once implemented at the CFIA. Other approaches that rely on the amplification and sequencing of mitochondrial markers [e.g., 6] or on the analysis of several microsatellite markers (e.g., [13]) have the potential of achieving levels of accuracy, resolution and scope similar to those of the present set of assays, but will require longer processing time. Conversely, the two AGM TaqMan assays developed earlier by other groups [14,24], although rapid, are limited in scope and subspecies resolution. With respect to cost, the suite of assays proposed here is relatively inexpensive to run (provided the necessary thermocycling equipment is available) and comparable in price to the sequencing of PCR products.

Several scientists have raised concerns about potential misidentifications when relying solely on mitochondrial markers to identify *L*. *dispar* strains [8,13,16,19,23,36]. Indeed, hybridization between *L*. *dispar dispar* and *L*. *dispar asiatica* can produce individuals displaying *L*. *dispar dispar* mitochondrial haplotypes with features of *L*. *dispar asiatica* nuclear genomes; in some cases, females of such insects have been observed to have strong flight capabilities [22]. Inclusion of an FS1-based assay in the procedure we developed was meant to flag such individuals, some of which may need to be considered AGM from a regulatory perspective. Most striking are individuals collected in central Asia that were identified as *L*. *dispar dispar* by *Simplex assay 3* but were homozygous for the Asian FS1 allele (Table 4; Fig 6). Using nine microsatellite markers, Wu et al. [13] showed clearly that gypsy moths from central Asia (Kazakhstan and Kyrgyzstan) have a mixed European and Asian genetic background, likely indicative of extensive hybridization at the boundary between the ranges of European and Asian populations. Interestingly, the original description of *L*. *dispar asiatica* was based on samples collected in Kazakhstan [37], pointing out potential ambiguities in subspecies assignment. Although gypsy moths from central Asia are much less likely to find their way into North America than their far eastern counterparts, due to differences in current trading pathways, any unknown sample displaying the EGM-COI/AA-FS1 genotype in *Simplex assay 1* and *Duplex assay 4* may need to be treated as AGM. It will not be possible to say with certainty whether such samples arose from populations with flight-capable females, but the outcome of these two tests should help narrow down the likely geographic origin of these moths. With respect to samples identified as heterozygous for the FS1 marker, an earlier study indicated that some European individuals with this genotype had flight-capable females [22]. These samples should therefore be treated with caution.

We believe that the TaqMan assays we developed have broad applicability and will be useful to regulatory agencies in any jurisdiction where invasive lymantriines are of concern. Minimal training is required for processing samples and diagnosis is made effortless if one uses the identification tool we provided (S2 File). Since this suite of assays is modular, users can also decide whether or not all modules need to be run for a given sample, providing some operational flexibility. The assay validation results we presented above indicate that a qPCR-based approach can provide both rapid and reliable identification of several invasive lymantriines. This, of course, does not preclude subsequent confirmation of species/subspecies identification through sequencing of the PCR products generated by the assays. In future work, we will seek to bring further improvements to our assay system, including the addition of markers that could provide greater resolution with respect to the identification of the geographic origins of unknown samples. Characterization of the genomic determinants of female flight capability could also be instrumental in the development of an assay module aimed at assessing this trait in unknown samples.

## Supporting Information

S1 File Details of primer and probe design, along with validation results for each individual TaqMan assay (1 assay/tab; first tab: general lymantriine primers). (XLSX)

S2 File Excel sheet tool for *Lymantria* species/subspecies identification. (XLSX)

S1 Table List of sources of DNA sequences used for assay development. (XLSX)

S2 Table Sequences of primers used for PCR amplification of potential markers. (DOCX)

## References

[1] Pogue MG, Schaefer PW. A review of selected species of *Lymantria* Hübner [1819] (Lepidoptera: Noctuidae: Lymantriinae) from subtropical and temperate regions of Asia, including the description of three new species, some potentially invasive to North America USDA Forest Service, Washington, DC; 2007.

[2] Dumouchel L. Plant health risk assessment: Asian gypsy moth. Canadian Food Inspection Agency, Plant Health Risk Assessment Unit, Science Branch, Ottawa, Canada. PHD request No. 99–24 UPD; 2010.

[3] USDA Pest alert—Asian gypsy moth. APHIS 81-35-027 leaflet. USDA Animal and Plant Health Inspection Service, Riverside Park, MD; 2016. Available online at: https://www.aphis.usda.gov/publications/plant_health/content/printable_version/fs_phasiangm.pdf

[4] Liebhold A, Mastro V, Schaefer PW. Learning from the legacy of Léopold Trouvelot. Bull Entomol Soc Am. 1989; 35: 20–22.

[5] Baranchikov YN. Ecological basis of the evolution of host relationships in Eurasian gypsy moth populations In: Wallner WE and McManus KA, tech. coords. Proceedings, Lymantriineae: A comparison of features of New and Old World tussock moth. USDA Forest Service, Northeastern Forest Experiment Station, Broomall, PA 1989 pp. 319–338.

[6] deWaard JR, Mitchell A, Keena MA, Gopurenko D, Boykin LM, Armstrong KF, et al Towards a global barcode library for *Lymantria* (Lepidoptera: Lymantriinae) tussock moths of biosecurity concern. PLoS ONE. 2010; 5: e14280 10.1371/journal.pone.0014280 21151562 PMC3000334

[7] CFIA List of pests regulated by Canada. Canadian Food Inspection Agency, Ottawa, Canada; 2016. Available online at: http://www.inspection.gc.ca/plants/plant-pests-invasive-species/pests/regulated-pests/eng/1363317115207/1363317187811

[8] Ball SL, Armstrong KF. DNA barcodes for insect pest identification: a test case with tussock moths (Lepidoptera: Lymantriineae). Can J For Res. 2006; 36: 337–350.

[9] Boykin LM, Armstrong KF, Kubatko L, De Barro P. Species delimitation and global biosecurity. Evol Bioinfo. 2012; 8: 1–37. 10.4137/EBO.S8532 PMC3256992 22267902

[10] Kang TH, Lee K-S, Lee H-S. DNA barcoding of the Korean *Lymantria* Hübner, 1819 (Lepidoptera: Erebidae: Lymantriinae) for quarantine inspection. J Econ Entomol. 2015; 108: 1596–1611. 10.1093/jee/tov111 26470300

[11] Bogdanowicz SM, Schaefer PW, Harrison RG. Mitochondrial DNA variation among worldwide populations of gypsy moths, *Lymantria dispar*. Mol Phylogenet Evol. 2000; 15: 487–495. 10860656 10.1006/mpev.1999.0744

[12] Chen F, Shi J, Luo Y-Q, Sun S-Y, Pu M. Genetic characterization of the gypsy moth from China (Lepidoptera, Lymantriineae) using inter simple sequence repeats markers. PLoS ONE. 2013; 8: e73017 10.1371/journal.pone.0073017 23951339 PMC3737146

[13] Wu Y, Molongoski JJ, Winograd DF, Bogdanowicz SM, Louyakis AS, Lance DR, et al Genetic structure, admixture and invasion success in a Holarctic defoliator, the gypsy moth (*Lymantria dispar*, Lepidoptera: Erebidae). Mol Ecol. 2015; 24: 1275–1291. 10.1111/mec.13103 25655667

[14] Qian L, An Y, Song J, Xu M, Ye J, Wu C, et al COI gene geographic variation of gypsy moth (Lepidoptera: Lymantriineae) and a TaqMan PCR diagnostic assay. DNA Barcodes. 2014; 2: 10–16.

[15] Armstrong KF, Ball SL. DNA barcodes for biosecurity: invasive species identification. Phil Trans R Soc B Lond. 2005; 360: 1813–1823. 10.1098/rstb.2005.1713 PMC1609225 16214740

[16] Chen F, Luo Y, Keena MA, Wu Y, Wu P, Shi J. DNA barcoding of gypsy moths from China (Lepidoptera: Erebidae) reveals new haplotypes and divergence patterns within gypsy moth subspecies. J Econ Entomol. 2015; 109: 366–374. 10.1093/jee/tov258 26371156

[17] Arimoto M, Iwaizumi R. Identification of Japanese *Lymantria* species (Lepidoptera: Lymantriineae) based on PCR–RFLP analysis of mitochondrial DNA. Appl Entomol Zool. 2014; 49: 159–169.

[18] Armstrong KF, McHugh P, Chinn W, Frampton ER, Walsh PJ. Tussock moth species arriving on imported used vehicles determined by DNA analysis. NZ Plant Prot. 2003; 56: 16–20.

[19] Bogdanowicz SM, Wallner WE, Bell J, Odell TM, Harrison RG. Asian gypsy moths (Lepidoptera: Lymantriineae) in North America: evidence from molecular data. Ann Entomol Soc Am. 1993; 86: 710–715.

[20] Garner KJ, Slavicek JM. Identification and characterization of a RAPD-PCR marker for distinguishing Asian and North American gypsy moths. Insect Mol Biol. 1996; 5: 81–91. 8673265 10.1111/j.1365-2583.1996.tb00043.x

[21] Harrison RG, ODell TM. Mitochondrial DNA as a tracer of gypsy moth origins, In: Wallner WE and McManus KA, tech. coords. Proceedings, Lymantriidae: a comparison of features of New and Old World tussock moths. USDA Forest Service, Northeastern Forest Experiment Station Broomall, PA; 1989 pp 265–273.

[22] Keena MA, Côté M-J, Grinberg PS, Wallner WE. World distribution of female flight and genetic variation in *Lymantria dispar* (Lepidoptera: Lymantriineae). Environ Entomol. 2008; 37: 636–649. 18559169 10.1603/0046-225x(2008)37[636:wdoffa]2.0.co;2

[23] Pfeifer TA, Humble LM, Ring M, Grigliatti TA. Characterization of gypsy moth populations and related species using a nuclear DNA marker. Can Entomol. 1995; 127: 49–58.

[24] Islam MS, Barr NB, Braswell WE, Martinez M, Ledezma LA, Molongoski J, et al A multiplex real-time PCR assay for screening gypsy moths (Lepidoptera: Erebidae) in the United States for evidence of an Asian genotype J Econ Entomol. 2015; 108: 2450–2457. 10.1093/jee/tov212 26453734

[25] Keena MA, Grinberg PS, Wallner WE. Inheritance of female flight in *Lymantria dispar* (Lepidoptera: Lymantriineae). Environ Entomol. 2007; 36: 484–494. 17445385 10.1603/0046-225x(2007)36[484:ioffil]2.0.co;2

[26] Owczarzy R, You Y, Groth CL, Tataurov AV. Stability and mismatch discrimination of locked nucleic acid-DNA duplexes. Biochemistry. 2011; 50: 9352–9367. 10.1021/bi200904e 21928795 PMC3201676

[27] Lumley LM, Cusson M. Linking adaptation, delimitation of evolutionarily significant units (ESUs), and gene function: a case study using hemlock looper ecotypes. Syst Entomol. 2013; 38: 428–439.

[28] Hajibabaei M, deWaard JR, Ivanova NV, Ratnasingham S, Dooh R, Kirk SL, et al Critical factors for the high volume assembly of DNA barcodes. Phil Trans R Soc B Lond. 2005; 360: 1959–1967. 10.1098/rstb.2005.1727 PMC1609220 16214753

[29] Ivanova NV, deWaard JR, Hebert PDN. An inexpensive, automation-friendly protocol for recovering high-quality DNA. Mol Ecol Notes. 2006; 6: 998–1002.

[30] deWaard JR, Ivanova NV, Hajibabaei M, Hebert PDN. Assembling DNA barcodes: Analytical protocols In: Cristofre M, editor. Methods in Molecular Biology: Environmental Genetics. Totowa, USA: Humana Press Inc; 2008 pp. 275–293. 10.1007/978-1-59745-548-0_15 18642605

[31] Hebert PDN, Cywinska A, Ball SL, deWaard JR. Biological identifications through DNA barcodes. Proc R Soc B Biol Sc. 2003; 270: 313–321. 10.1098/rspb.2002.2218 PMC1691236 12614582

[32] CCDB, the Canadian Centre for DNA Barcoding. 2013. Available online at: http://www.ccdb.ca/resources.php

[33] Holland PM, Abramson RD, Watson R, Gelfand DH. Detection of specific polymerase chain reaction product by utilizing the 5'——3' exonuclease activity of *Thermus aquaticus* DNA polymerase. Proc Natl Acad Sci U S A. 1991; 88: 7276–7280. 1871133 10.1073/pnas.88.16.7276 PMC52277

[34] Newton CR, Graham A, Heptinstall LE, Powell SJ, Summers C, Kalsheker N, et al Analysis of any point mutation in DNA. The amplification refractory mutation system (ARMS). Nucleic Acids Res. 1989; 17: 2503–2516. 2785681 10.1093/nar/17.7.2503 PMC317639

[35] Rutledge RG. A Java program for LRE-based real-time qPCR that enables large-scale absolute quantification. PLoS ONE. 2011; 6: e17636 10.1371/journal.pone.0017636 21407812 PMC3047581

[36] Bogdanowicz SM, Mastro VC, Prasher DC, Harrison RG. Microsatellite DNA variation among Asian and North American gypsy moths (Lepidoptera: Lymantriineae). Ann Entomol Soc Am. 1997; 90: 768–775.

[37] Korb SK, Pozhogin DA. A new species of *Lymantria* Hübner [1819] from South-East Kazakhstan with some notes on the systematics of *Lymantria dispar* (Linnaeus, 1758) (Lepidoptera: Lymantriineae). Eversmannia. 2012; 31/32: 62–66.

