# Supplementary material for: A Multi-Species TaqMan PCR Assay for the Identification of Asian Gypsy Moths (Lymantria spp.) and Other Invasive Lymantriines of Biosecurity Concern to North America
Source: PLoS One. 2016 Aug 11;11(8):e0160878. doi: 10.1371/journal.pone.0160878 (PMC4981461; doi:10.1371/journal.pone.0160878)
Supplement: S2 Table — (DOCX) [file pone.0160878.s004.docx]

**Supplementary Table 2.** Primers used for sequencing

| Target | Primer name | Sequence (5’→ 3’) | Amplicon length (bp) |
| --- | --- | --- | --- |
|  |  |  |  |
| COI-5P | LEPF/LepF1 | ATTCAACCAATCATAAAGATATTGG | 657 |
|  | LEPR/LepR1 | TAAACTTCTGGATGTCCAAAAAATCA |  |
|  | MLepF1 | GCTTTCCCACGAATAAATAATA | 660 |
|  | MLepR1 | CCTGTTCCAGCTCCATTTTC |  |
|  | COI_F (LC01490) | GGTCAACAAATCATAAAGATATTG | 707 |
|  | COI_R (HC02198) | TAAACTTCAGGGTGACCAAAAAATCA |  |
|  |  |  |  |
| COI-3P | COI-3P FSEQ 2041-2069 | GGTGCTATTACWATATTATTAACTGACCG | 906 |
|  | COI-3P RSEQ 2979-3008 | GGGTTTAAATCCATTACATATAATCTGCCA |  |
|  | COI-3P RSEQ 2332-2354 | GCAGAGGTAAAGTAAGCTCGTGT | 313^a^ |
|  |  |  |  |
| ATP6-ATP8 | ATP6/ATP8-F | GCGGAACTAACCACAGATTT | 960 |
|  | ATP6/ATP8-R | TGATTGGATAACCGCAACTG |  |
|  |  |  |  |
| COII | Lym_COII_SEQ_F38-62 | TGGATTTAAACCCCATTYATAAAGG | 608 |
|  | Lym_COII_SEQ_R645-70 | GAACATTGACCAWAAAAAATTCCWGG |  |
|  | S2994F_COII | GAACATTCITATAATGAACTYCCT | 766 |
|  | A3772R_COII | GAGACCATTACTTGCTTTCAGTCATCT |  |
|  |  |  |  |
| Cytb | REVCB2H | TGAGGACAAATATCATTTTGAGGW | 570 |
|  | REVCBJ | ACTGGTCGAGCTCCAATTCATGT |  |
|  |  |  |  |
| EF1α | EF1_Starsky_For | CACATYAACATTGTCGTSATYGG | 541 |
|  | EF1_Luke_Rev | CATRTTGTCKCCGTGCCAKCC |  |
|  |  |  |  |
| ITS2 | ITSF_Barr_857-881 | TTGAACATCGACATTTCGAACGCAC | 633 |
|  | ITSR_Barr_1470-1489 | TCCTCCGCTTATTGATATGC |  |
|  |  |  |  |
| ND1 | ND1-F2 | AGCAAAAGCTAATACAGGAG | 276 |
|  | ND1-R2 | ATGGATTTACTATTTTGGCAG |  |
|  |  |  |  |
| ND2 | ND2-F | TGGATGTTGAATTGGGTTAGA | 950 |
|  | ND2-R | ATTGCAAATTTTAAGGAGTATTT |  |
|  | ND2-ANF1 | TTGATTCCCCAATATTGTTGAAGGAT | 429 |
|  | ND2-ANR1 | AATCCAATAAATGGGGGTAATCCTCCT |  |
|  |  |  |  |
| ND6-Cytb | ND6/cytb-F | AACCCCCTTTCTATAGGATT | 960 |
|  | ND6/cytb-R | TGATCCAGTTTGATGAAGAA |  |
|  |  |  |  |

^a^ For *L. postalba* and *L. albescens* samples; COI-3P FSEQ 2041-2069/COI-3P RSEQ 2332-2354
